# Episodes of gene flow and selection during the evolutionary history of domesticated barley

**DOI:** 10.1186/s12864-021-07511-7

**Published:** 2021-04-01

**Authors:** Peter Civáň, Konstantina Drosou, David Armisen-Gimenez, Wandrille Duchemin, Jérôme Salse, Terence A. Brown

**Affiliations:** 1 Department of Earth and Environmental Sciences, Manchester Institute of Biotechnology, University of Manchester, Manchester, M1 7DN UK; 2 INRA-Université Clermont-Auvergne, UMR 1095 GDEC, 5 Chemin de Beaulieu, 63000 Clermont-Ferrand, France; 3 KNH Centre for Biomedical Egyptology, Faculty of Biology, Medicine and Health, University of Manchester, 99 Oxford Road, Manchester, M13 9PG UK; 4 Institut de Génomique Fonctionnelle de Lyon, Université de Lyon, 69364 Lyon, France; 5 Center for Scientific Computing (sciCORE), University of Basel, Basel, Switzerland

**Keywords:** Barley, Exome sequences, Fertile Crescent, *Hordeum vulgare*, Gene flow, Origins of agriculture, Selection, Selective sweep

## Abstract

**Background:**

Barley is one of the founder crops of Neolithic agriculture and is among the most-grown cereals today. The only trait that universally differentiates the cultivated and wild subspecies is ‘non-brittleness’ of the rachis (the stem of the inflorescence), which facilitates harvesting of the crop. Other phenotypic differences appear to result from facultative or regional selective pressures. The population structure resulting from these regional events has been interpreted as evidence for multiple domestications or a mosaic ancestry involving genetic interaction between multiple wild or proto-domesticated lineages. However, each of the three mutations that confer non-brittleness originated in the western Fertile Crescent, arguing against multiregional origins for the crop.

**Results:**

We examined exome data for 310 wild, cultivated and hybrid/feral barley accessions and showed that cultivated barley is structured into six genetically-defined groups that display admixture, resulting at least in part from two or more significant passages of gene flow with distinct wild populations. The six groups are descended from a single founding population that emerged in the western Fertile Crescent. Only a few loci were universally targeted by selection, the identity of these suggesting that changes in seedling emergence and pathogen resistance could represent crucial domestication switches. Subsequent selection operated on a regional basis and strongly contributed to differentiation of the genetic groups.

**Conclusions:**

Identification of genetically-defined groups provides clarity to our understanding of the population history of cultivated barley. Inference of population splits and mixtures together with analysis of selection sweeps indicate descent from a single founding population, which emerged in the western Fertile Crescent. This founding population underwent relatively little genetic selection, those changes that did occur affecting traits involved in seedling emergence and pathogen resistance, indicating that these phenotypes should be considered as ‘domestication traits’. During its expansion out of the western Fertile Crescent, the crop underwent regional episodes of gene flow and selection, giving rise to a modern genetic signature that has been interpreted as evidence for multiple domestications, but which we show can be rationalized with a single origin.

**Supplementary Information:**

The online version contains supplementary material available at 10.1186/s12864-021-07511-7.

## Background

Although almost all of human subsistence depends on domesticated plants and animals, the genetics of domestication often remains obscure. A case in point is cultivated barley (*Hordeum vulgare* subs. *vulgare*, the domesticated form of wild *H. vulgare* subsp. *spontaneum*), which is the fifth most-grown crop worldwide [1] and is mainly used for animal fodder and beer brewing. Alongside diploid and tetraploid wheat, barley is one of the crops that founded the Neolithic transition in the Fertile Crescent, some 10,000 years ago [2].

The cultivated and wild subspecies of barley remain remarkably similar and most phenotypic novelties are not universally present in modern cultivars. The sole trait shared by all domesticated varieties and strictly differentiating wild and cultivated forms is ‘non-brittleness’ of the rachis (the stem of the inflorescence) at maturity, which facilitates harvest in agricultural settings but hinders seed dispersal in nature. Early genetic studies revealed that non-brittleness in barley is determined by either of two linked loci, *Btr1* and *Btr2* [3], prompting suggestions that barley was domesticated at least twice. This hypothesis was supported by an observed gradient of haplotype frequencies along the east-west axis, which was interpreted as indicating domestications in the Fertile Crescent and in a region west of the Zagros mountains [4, 5], and has more recently been used as evidence that Tibet was a possible domestication centre [6, 7]. Comparisons of nuclear and plastid markers have also led to the suggestion that barley could have been domesticated in the Horn of Africa [8]. However, analysis of large-scale genomic datasets has failed to identify linear relationships between multiple wild and domesticated groups, and instead places all cultivars in a single cluster [9, 10]. More detailed studies of the *Btr* loci have also argued against distinct western and eastern origins for cultivated barley. Three causal non-brittleness *btr* mutations have now been identified [11, 12], but the genealogy of each of these points to an origin in the western arm of the Fertile Crescent. As hypotheses proposing multiregional independent domestications of barley have become increasingly difficult to rationalize with different lines of evidence, some studies now conclude that the ancestry of barley is ‘mosaic’, resulting from genetic interaction between multiple wild or proto-domesticated lineages [9, 13].

Other than non-brittleness, no other recognized phenotypic difference universally separates cultivated and wild barley: traits such as photoperiod insensitivity, absence of the vernalization requirement, six-rowed seedheads and naked grain have not been fixed during domestication and appear to result from facultative or regional selective pressures [14–16]. Does this mean that in barley a single phenotypic change (i.e. non-brittleness) is required for successful domestication, or are there other universal, yet to be discovered biological features required for efficient cultivation? And if non-brittleness – achieved by three alternative mutations – is indeed the only genetic prerequisite for cultivation, does this mean that there is no selection history shared by all extant barley cultivars? These questions have profound importance for our understanding of early agriculture and the genetics of barley domestication, but they have not been satisfactorily answered to date.

Identification of adaptive genes without prior knowledge of the phenotypes they confer is possible with a bottom-up approach that begins with population genetic screening to detect signatures of selection [17]. For barley, this approach has previously been attempted on a genome-wide scale [10], but yielded only two statistically significant signals, one associated with the *Btr1/Btr2* region of chromosome 3H, and a second on chromosome 1H, in a region that apparently did not contain candidate domestication loci. Another recent study identified dozens of candidate genes potentially involved in domestication, but the screening was limited to 1666 pre-selected loci [9]. Importantly, neither of these studies performed scans specifically on the domesticated subpopulations on which selection is likely to have operated.

The availability of exome capture datasets for multiple barley accessions, coupled with improvements in the barley reference genome, enable the evolutionary history of cultivated barley to be examined in greater detail. We therefore analysed exome data for 310 wild, cultivated and hybrid/feral barley accessions in order to delineate the demographic history of domesticated barley, and subsequently to identify signatures of selection in genetically-defined domesticated groups, as well as the inter-group overlaps of these signatures and the candidate gene variants that were targeted during domestication.

## Results and discussion

### Population history of cultivated barley

Exome data for 112 wild barley accessions, 15 hybrid or feral lines, and 183 landraces and improved cultivars (Additional file 1: Table S1), including a 6000 years old specimen [18] serving as a temporal reference of the domestication process (referred to as the ‘6ky barley’), were mapped against the pseudomolecule-level assembly of the barley genome [19]. The wild accessions were divided into four populations – western Fertile Crescent, eastern Fertile Crescent, Mediterranean, and Central Asia – based on their collection points (see Methods).

**Fig. 1:**
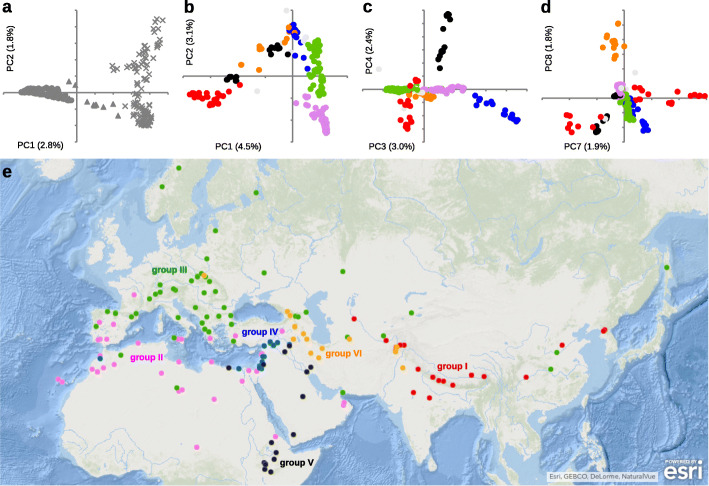
Structure and geography of barley populations. **a** The top two PCs of the nucleotide diversity in wild and cultivated barley. Wild barley accessions are marked as crosses and domesticated accessions as full circles. Several accessions previously described as wild, but collected outside of the primary distribution range, were labelled as feral/hybrid (full triangles) and excluded from further analyses (see Additional file 1: Table S1) . None of the top 20 PCs placed cultivated barley in separate clusters (not shown). **b**–**d** PCAs of the cultivated barleys (wild barley excluded). On all three panels, group membership is indicated with the same colours as on the map below. **e** Geography of the domesticated groups defined by the PCA of cultivated barley

Principal component analysis (PCA) of nucleotide diversity placed all cultivated barley in a single cluster separated from the wild subspecies (Fig. 1a), mirroring the pattern reported previously [9, 10]. When only the diversity of cultivated barley is analysed by the PCA, multiple clusters or ‘groups’ can be identified (Fig. 1b–d). From the information provided by PCs 1–8 (see Methods), 95% of the cultivated accessions could be assigned to six population genetic groups (Fig. 1e). Four of these – eastern (group I), Mediterranean (II), central European (III) and Arabian-Ethiopian (V) – are consistent with genetic clusters identified in a different dataset [13]. The additional two groups are a cluster of two-rowed landraces mostly located in the Fertile Crescent (group IV), and a cluster predominantly comprising landraces from Transcaucasia and Iran (group VI). The Arabian-Ethiopian group V can also be further subdivided into Va (Ethiopia) and Vb (Western Asia), but these accessions were retained as a single group in most subsequent analyses due to the small sample size. The population structure inferred from the PCAs was supported by a distance-based Neighbour-Net analysis [20] (Additional file 2: Fig. S1), in which each group formed a single cluster, with the exception of groups Va and Vb which were positioned at different places in the graph. The population structure was also supported by the ancestry coefficients obtained by sNMF analysis [21] (Additional file 3: Fig. S2), which differentiated groups I–III at *K* = 4 and the remaining groups at *K* = 7, with extensive admixture apparent at all *K* values.

**Fig. 2:**
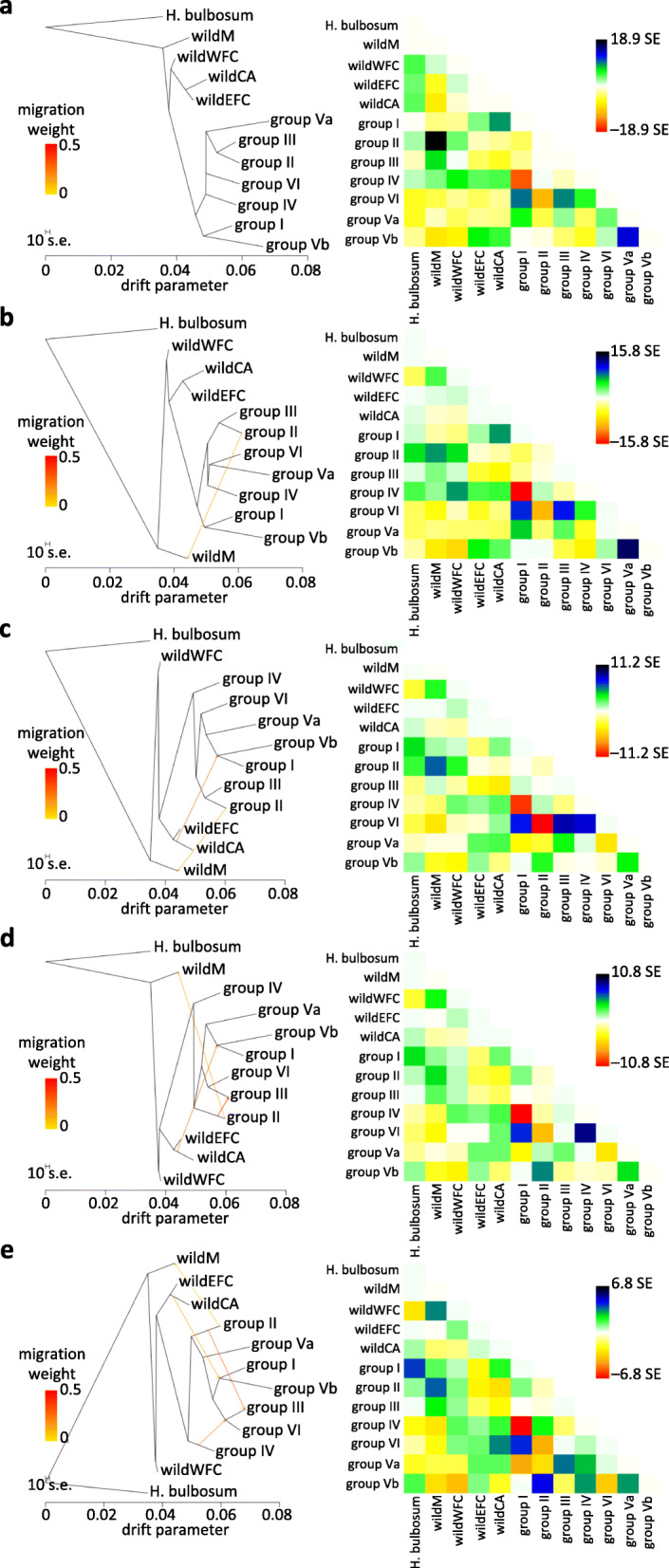
Inference of population splits with various numbers of population mixtures. TreeMix population graphs (left) and the residual matrices (right) are shown for modelling **a** zero migration (admixture) events, **b** 1 event, **c** 2 events, **d** 3 events, and **e** 4 events. Admixture is indicated by arrows that are coloured according to the inferred relative genetic contribution. All shown admixture edges improve the fit of the graphs to the data with the highest significance (*p* < 2.22507 × 10^− 308^), except the group II → group III migration on the panel d (2.10942 × 10^−15^), and the group IV → (groupIII, groupVI) migration on the panel e (1.11022 × 10^−16^). The residual matrices quantify the inter-group covariance of allelic frequencies not captured by the respective graphs, and thus indicate pairs of populations where additional gene flow edges might improve the fit

To examine the relationships between the populations in greater detail, we used TreeMix [22], a statistical model that enables chronological population splits and mixtures to be inferred from the covariance of allele frequency data. The results were consistent with the diversity patterns described above, and suggested that all cultivated barley has a common base and the six extant groups are the result of population splits and significant admixture events (Fig. 2). The Mediterranean wild barleys represent the most ancient split. The cultivated barleys are always resolved as a single cluster, and when 2–4 admixture events are modelled the oldest branch is formed by the Fertile Crescent group IV, which consists of two-rowed landraces and includes the 6ky barley excavated in Israel. Two episodes of genetic exchange between wild and domesticated populations are consistently identified in the Treemix analyses. The first of these is between the Mediterranean wild population (Cyprus, Greece and Libya) and the Mediterranean domesticated group II, with additional exchange between group II and central European group III. The second exchange is between the Central Asian wild population and Transcaucasian-Iranian group VI and the Arabian group Vb.

We also employed the ABBA-BABA test [23] to further investigate the pattern of gene flow between populations (Table 1). The ABBA-BABA test and its associated statistics *D* (Patterson’s *D*) and *f* (*f*_*G*_, *f*_*d*_) were developed to detect and quantify introgressions in rooted four-taxon sets [23, 24], and the concept can be extended to detect admixture among populations. If an ancestral allele (defined by an outgroup) at a biallelic locus is designated ‘A’ and a derived allele is designated ‘B’, then three populations and their outgroup with the relationship (((P1, P2), P3), O) will show a relatively high amount of the ‘BBAA’ pattern (i.e. where the derived alleles are shared among the sister populations P1 and P2). Patterns where derived alleles are shared by non-sister groups (i.e. ‘ABBA’ and ‘BABA’) occur less frequently (given a correct underlying tree), and should be in equal proportions under a neutral coalescent model of evolution. An excess of shared derived alleles indicated by the relative abundance of the ABBA or BABA patterns is then commonly interpreted as a consequence of gene flow between P2 and P3, or P1 and P3, respectively. However, the test becomes more complex when the number of populations involved in the analysis is high and their hierarchical relationship is unclear. Here, six cultivated groups (I–VI) and two wild superpopulations east and west of the Euphrates (corresponding to the major split on the Neighbor-Net network, Additional file 2: Fig. S1) create 56 different four-taxon subsets with a fixed outgroup (i.e. all combinations of three populations out of eight). Since quantification of the ABBA and BABA patterns is only meaningful on a ‘correct’ four taxon tree, the major underlying tree topology needs to be known a priori or identified based on the BBAA counts [25]. Here we followed the latter approach; for any combination of three populations with an outgroup, we identified the major tree topology as the one with the highest count of BBAA patterns. Those four-taxon sets that contain three cultivated populations and no wild population are uninformative in respect to the domestication of barley. Therefore, Table 1 shows only the four-taxon sets with one or two cultivated groups, their ‘correct’ tree based on the BBAA counts, and the associated statistics of gene flow. In the four-taxon sets that featured exactly one domesticated group, the eastern wild superpopulation is resolved as sister to the cultivated groups I, V and VI, while the western wild superpopulation is sister to the cultivated groups II, III and IV. This indicates biphyletic origins for cultivated barley, without significant gene flow. However, all four-taxon sets that featured exactly two domesticated groups always resolved those two groups as sisters in the major topologies. This suggests a single origin for all cultivated barley, with additional significant gene flow. These major topologies represent a collection of mutually incompatible partial trees. The first scenario (two origins without gene flow) is incompatible with all partial trees where either of the (I, V, VI) and (II, III, IV) groups are sisters; however, the second scenario (single origin with significant gene flow) can be reconciled with all partial trees, and is therefore the logical conclusion of the ABBA-BABA tests.

**Table 1:** ABBA-BABA-related statistics

Four-taxon set	Best tree (according to the BBAA count with fixed outgroup)	D-statistics (excess of ABBA patterns)	fG (genomic fraction shared through gene flow)	Gene flow significance (FWER correction) * *p* < 0.05, ** *p* < 0.001
One domesticated group	(((wild-W, group IV), wild-E), O)	0.0198	0.0202	
	(((wild-E, group VI), wild-W), O)	0.0132	0.0171	
	(((group I, wild-E), wild-W), O)	0.0118	0.0177	
	(((group II, wild-W), wild-E), O)	0.0133	0.016	
	(((group V, wild-E), wild-W), O)	0.0043	0.0069	
	(((group III, wild-W), wild-E), O)	0.0053	0.0068	
Two domesticated groups	(((group II, group V), wild-E), O)	0.0586	0.0614	**
	(((group III, group V), wild-E), O)	0.0501	0.0543	**
	(((group I, group IV), wild-W), O)	0.0374	0.0501	**
	(((group II, group I), wild-E), O)	0.0552	0.0610	**
	(((group I, group VI), wild-W), O)	0.0321	0.0386	**
	(((group II, group VI), wild-E), O)	0.0395	0.0404	**
	(((group III, group I), wild-E), O)	0.0468	0.0545	*
	(((group II, group IV), wild-E), O)	0.0430	0.0438	*
	(((group III, group VI), wild-E), O)	0.0322	0.0321	*
	(((group V, group IV), wild-W), O)	0.0315	0.0414	*
	(((group V, group VI), wild-W), O)	0.0233	0.0288	*
	(((group I, group II), wild-W), O)	0.0290	0.0365	*
	(((group V, group II), wild-W), O)	0.0214	0.0268	
	(((group I, group III), wild-W), O)	0.0287	0.0342	
	(((group V, group III), wild-W), O)	0.0210	0.0242	
	(((group III, group IV), wild-E), O)	0.0331	0.0353	
	(((group VI, group I), wild-E), O)	0.0202	0.0217	
	(((group VI, group V), wild-E), O)	0.0207	0.0219	
	(((group II, group III), wild-E), O)	0.0119	0.0101	
	(((group IV, group I), wild-E), O)	0.0154	0.0175	
	(((group III, group IV), wild-W), O)	0.0119	0.0170	
	(((group VI, group IV), wild-W), O)	0.0102	0.0129	
	(((group IV, group V), wild-E), O)	0.0166	0.0177	
	(((group II, group IV), wild-W), O)	0.0106	0.0141	
	(((group I, group V), wild-W), O)	0.0095	0.0115	
	(((group III, group VI), wild-W), O)	0.0022	0.0029	
	(((group VI, group IV), wild-E), O)	0.0032	0.0033	
	(((group III, group II), wild-W), O)	0.0013	0.0016	
	(((group II, group VI), wild-W), O)	0.0008	0.0011	
	(((group V, group I), wild-E), O)	0.0003	0.0003	

*Abbreviations*: *wild-E* wild superpopulation east of the Euphrates, *wild-W* wild superpopulation west of the Euphrates

**Fig. 3:**
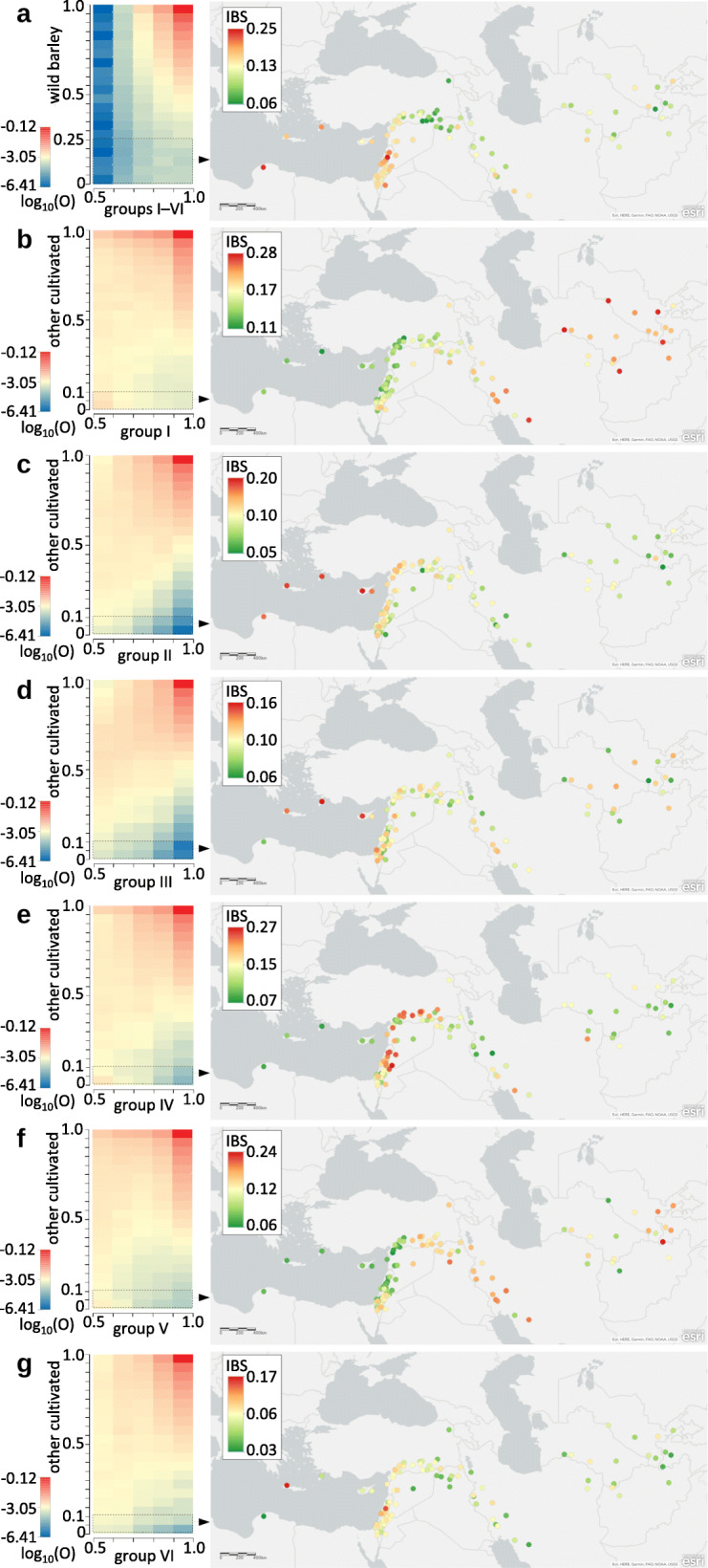
Ancestry-informative SNPs in the exome data. The left panels present the distribution of SNPs on joint allele frequency spectra, and delineate ancestry-informative frequency classes (dashed lines). Observed proportions of variants in each frequency class (O) were logarithmically transformed and expressed by a colour gradient. The right panels show similarity of wild accessions to the selected variant sets, measured as identity-by-state (IBS). **a** Variants with frequencies > 0.5 in all cultivated groups and their frequency distribution in wild barley (left). The dashed line delineates 5666 ancestry-informative variants and their occurrence in wild accessions is depicted on the map (right). Note that although the allele frequency spectrum shows allele frequencies for the entire cultivated supergroup, we only selected variants that are truly major in each of groups I–VI. **b** Frequency distribution of major group I variants in the remaining cultivated population (left) and occurrence of the selected ancestry-informative alleles in wild accessions (right). **c**–**g** Equivalent description of major variants in groups II–VI, respectively

We have previously highlighted problems with the use of analytical methods that assume a tree- or pseudo-tree like structure in studying population histories that are reticulated rather than tree-like, due to gene flow and hybridization between lineages [26, 27]. To quantify signals of ancestry directly from the exome data, without a priori assumptions of the nature of inter-population relationships, we therefore characterized sets of ancestry-informative variants (Fig. 3). Within the base dataset (see Methods) consisting of 2,595,471 single nucleotide polymorphisms (SNPs), all six cultivated groups share an identical major allele (allelic frequency *p* > 0.5) at 2,284,720 sites (88%), indicating relatively low inter-group differentiation. The vast majority of these variants are also present in wild barley at high frequencies and are therefore uninformative for tracing the origin of the common genomic fraction. In contrast, those variants that are major in all cultivated groups while relatively rare in wild barley (*p* ≤ 0.25) are ancestry-informative, and these have the highest concentration in the wild accessions collected from the western arm of the Fertile Crescent and Libya (Fig. 3a). A potentially confounding factor here is the possibility of wild genomes being admixed with cultivated barley post-domestication. Indeed, the Libyan wild accession carrying a high proportion of these ancestry-informative variants has been previously shown to have the domesticated *btr2* allele [12], suggesting past introgressions from barley cultivars. These data therefore indicate that the western Fertile Crescent is the source of the genomic fraction common to all groups of cultivated barley. Interestingly, wild accessions from south-eastern Turkey east of Euphrates, the assumed area of einkorn and emmer domestication [28, 29], are among the least similar to the common barley fraction (Fig. 3a).

In contrast to the high number of variants shared by all cultivated groups at high frequencies, group-specific (or private) variants are relatively scarce on the genome-wide scale. For each group, we quantified major alleles (*p* > 0.5) that are rare (*p* ≤ 0.1) in all other cultivated groups (Fig. 3b-g). The eastern group I has the highest number of this class of variants (12,251; 0.47% of all sites), and their distribution in wild accessions indicates a central Asian origin (Fig. 3b). The Mediterranean group II has 2355 alleles of this class (0.09% of all sites) appearing mainly in wild barley from Crete, Rhodes, Cyprus and Libya (Fig. 3c). The central European group III has only 1509 such alleles (0.06% of all sites), with distribution in wild barley similar those in the group II (Fig. 3d). Major alleles of the Fertile Crescent group IV that are rare in the other cultivated groups (7330; 0.28% of all sites) are most frequent in the Levant and south-eastern Turkey (Fig. 3e). For the Arabian-Ethiopian group V, this fraction (5974; 0.23% of all sites) points to the eastern arm of the Fertile Crescent and Central Asia (Fig. 3f), while such variants of group VI from Transcaucasia and Iran (2922; 0.11% of all sites) are mostly found in two geographically distant wild accessions (Fig. 3g).

**Fig. 4:**
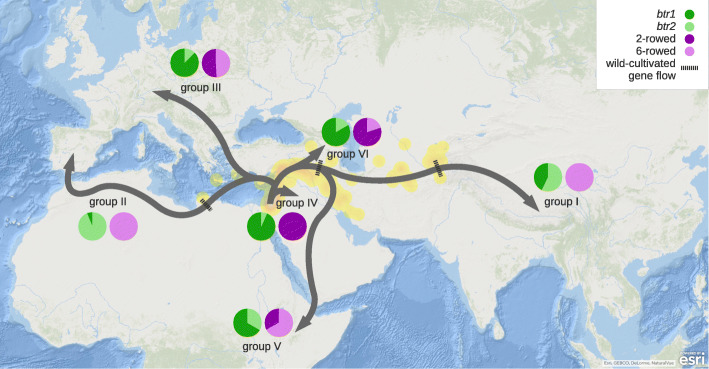
Population history of cultivated barley. Geographical summary of the population history reconstructed from all collected evidence. The pie charts indicate the proportions within each group of the indehiscence alleles *btr1* and *btr2* (green; see Additional file 4: Fig. S3 for details) and of 2- and 6-rowed barleys (purple). The natural distribution range of wild barley is approximated with yellow shading. The likely locations of the inferred gene flow between wild and cultivated barley are indicated by dashed lines

In summary, analysis of the exome data set indicates that cultivated barley is structured into six genetically-distinct groups (PCAs, Fig. 1; Neighbour-Net, Additional file 2: Fig. S1) that display admixture (sNMF, Additional file 3: Fig. S2), resulting at least in part from two or more significant passages of gene flow (Treemix, Fig. 2) during descent from a single founding population (ABBA-BABA, Table 1) that was located in the western arm of the Fertile Crescent (Treemix, Fig. 2; ancestry-informative variants, Fig. 3). In the most likely interpretation of these results, the initial expansion of barley cultivation split the Fertile Crescent population into western and eastern branches (Fig. 4). The western branch (the Mediterranean and European groups II and III, corresponding to the traditional southern and central European trajectories for the spread of agriculture into Europe [30]) engaged in mutual genetic exchange with wild populations in Libya and the islands of the eastern Mediterranean. The eastern branch split to give the domesticated populations of Central Asia (group I) and Ethiopia-Arabia (group V), admixture with central Asian wild barleys occurring before and possibly after this split. Our results therefore contradict previously published scenarios of geographically separate domestications [4–8], but are in agreement with the genealogy of the *Btr* loci [11, 12], which indicate that although multiple *btr* mutations were selected to alter the quality of the rachis, these events were geographically close and operated on the same founding population. It is noteworthy that the wild population in the western arm of the Fertile Crescent harbours the greatest diversity (Additional file 2: Fig. S1 & Additional file 5: Fig. S4). The high diversity of the founding population, coupled with recombination in the early fields, is likely responsible for the mosaic ancestry patterns reported in domesticated barley [13].

It is important to stress that, from the anthropological perspective, the existence of a single founding population for cultivated barley does not necessarily equate with a single ‘domestication event’. It is conceivable that establishment of this initial (pre) domesticated population in the Fertile Crescent involved independent sampling of wild plants by pre-farming groups, regionally dispersed origins of barley cultivation with parallel selection pressures, and/or mixing of cultivated populations. The cultivated gene pool was then further enriched by hybridizations with distinct wild populations, once cultivation spread outside of the Fertile Crescent. These genetic interactions rather than independent domestications are the main source of the distinctiveness between the western and eastern barley cultivars, further amplified by selection, as detailed below.

### Patterns of selection during barley domestication

**Fig. 5:**
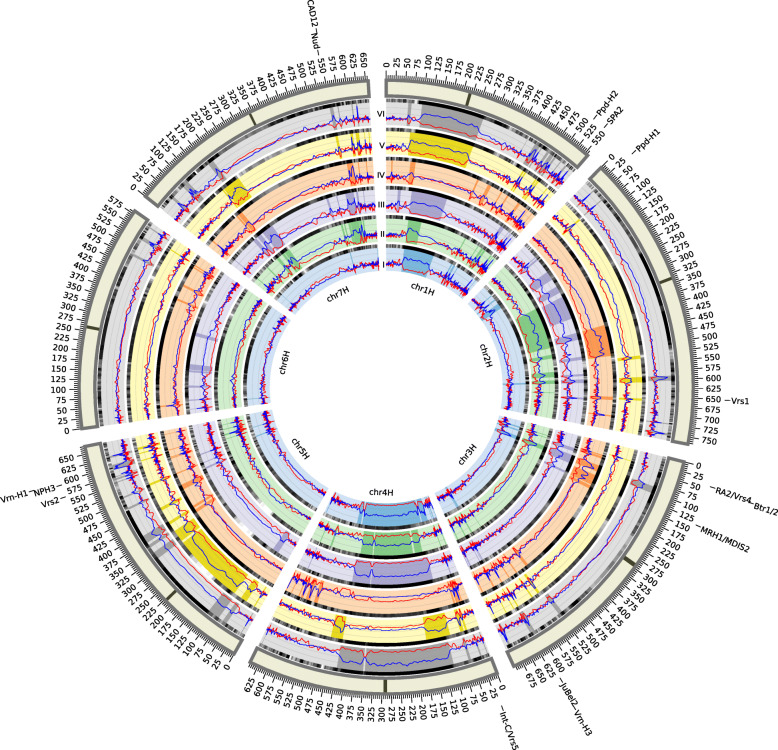
Selective sweeps within the barley genomes. The barley genome, comprising chromosomes 1H–7H, is presented in a circular layout [31] with a different concentric track for each of the geographically-defined groups I–VI. Within each track, the values for Tajima’s D and the diversity reduction index [log_2_(DRI)] are indicated by the red and dark blue plots, respectively, sharing the y-axis with a range [− 3, 9]. The identified sweep regions are highlighted. The degree of similarity to the 6ky barley genome is indicated at the outer edge of each track by the greyscale line (gradation from white for < 83% similarity to black for > 99% identity, calculated for each genomic window as the proportion of major alleles matching the 6ky variants). In the outer track each chromosome is represented as a box, with the centromere indicated by the crossbar and the physical coordinates (Mb) marked. Positions of previously described domestication genes [15, 16] and the genes with protein-changing variants identified in this study (see Table 2) are shown in the outermost track

**Fig. 6:**
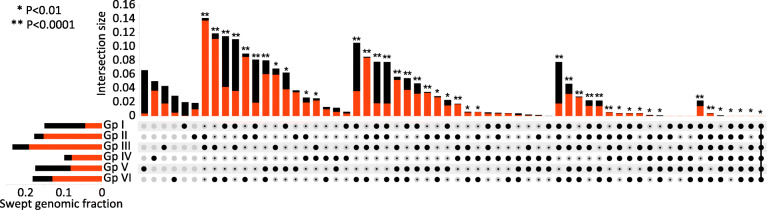
UpSet-style plot [34] summarizing the swept genomic regions and their intersection sizes. The black/red bars on the left indicate the portion of the genome that was classified as hard sweeps in each of the six cultivated groups (e.g. ~ 15% of the genome [0.15 genome fraction] is swept in group I). The main graph then provides details of the components of these sweeps that are group-specific or shared with one or more other groups. The y-axis indicates the fraction of the reference genome, and the graphics under the x-axis reveal the group(s) with which each fraction is associated. The first six columns show how much of the genome is covered by group-specific hard sweeps. For example, column 1 shows that group V-specific sweeps have an intersection size of 0.066 and hence cover 6.6% of the reference genome. The subsequent columns show the sizes of the aggregated intersections. For example, column 7 shows that the spatial overlap between the sweeps in groups II and III (indicated by black circles) and any other group that intersects this overlap (indicated by the black dots within the grey circles) has an intersection size of 0.141 and so covers 14.1% of the reference genome. The asterisks above the columns indicate those intersection sizes that are significantly larger than a stochastic overlap of independent sweeps, due to shared selection history or parallel targeting. Conversely, absence of significance indicates that intersections of the same or smaller size could occur simply by chance with the given number and length of independent sweeps. Throughout the Figure, red is used to show those fractions with > 99% sequence similarity to the 6ky barley. For example, almost all the sequences simultaneously swept in groups II and III (column 7) have > 99% identity to the corresponding sequences in the 6ky barley. The last column, on the extreme right, shows the size of the regions that are simultaneously swept in all six groups. Although this region represents only 0.005% of the reference genome, it is still significantly larger than would be expected by chance. Its similarity to the 6ky barley is > 99%, indicating early selection of this sequence

To understand the patterns of selection occurring during barley domestication, we used an optimized approach for detection of selective sweeps in barley exome data (Additional file 6: Supplementary Note). Diversity metrics were scanned across all chromosomes, and in each of the six groups those regions with severely reduced nucleotide diversity were identified (Fig. 5). These are likely to correspond to hard sweeps, which arise when strong selection is applied on a variant with low initial frequency (possibly a novel mutation), and the hitch-hiking effect depletes genetic diversity in the surrounding region [33]. Since domestication variants are expected to follow such a scenario (rare or absent in the wild superpopulation, reaching fixation in cultivated groups), we focused on these hard sweeps and employed a stringent detection threshold. A specific selection pattern was recovered for each group, consisting of 29–61 sweeps with median length 4.3 Mb that cover 10–23% of the genome (highlighted in Fig. 5). These sweeps, their intersections among the groups, and their similarity to the 6ky barley provide information on the chronology of domestication against the background of the population splits and mixtures (Fig. 6). The oldest, Fertile Crescent-bound group IV appears rather distinct from the other groups, which is in part due to its modest sweep lengths. The other groups have generally fewer, but much larger sweeps, which can be explained by limited opportunities for breaking the linkage blocks by recombination in new geographic areas. The majority of sweeps in the Mediterranean and European groups (and 97% of the sweeps common to both) carry haplotypes that were already present in the 6ky barley, indicating that the western population branch stems from the Fertile Crescent, but encountered further reductions of diversity, presumably linked to environmental adaptation and/or human selection for desirable phenotypes. Interestingly, the group-specific sweeps in the eastern population branch (groups I, V and VI) carry haplotypes that are largely absent from the 6ky barley, suggesting that the gene flow detected in the genealogy of the eastern barley contributed to domestication by providing valuable new variants. Moreover, the sweep intersections between the eastern group I and groups II, III and VI are large but often distinct, indicating parallel selection in the east and the west acting on the same loci with different haplotypes.

A conspicuous feature of the selective sweeps is their low inter-group sharing. For example, only 0.6% of the group I and IV genomes are swept simultaneously, an amount which could have occurred without any shared selection history simply by chance (Fig. 6). Consequently, although almost half of the barley genome (47.8%) is classified as swept in at least one of the cultivated groups, only 3.6% of the genome is swept when all groups are combined in a balanced manner (Additional file 7: Fig. S5). This confirms that selection in barley largely followed region-specific pathways. When combined with gene flow with local wild populations, these selection events would provide cultivated barley with group-specific genetic features that could be mistaken as evidence for multiple domestication centres [4–8]. None of the previously described barley domestication genes [15, 16] are swept in all six groups and some are not swept in any group (Fig. 5 & Additional file 8: Table S2). This is unsurprising, since traits such as photoperiod sensitivity and spike architecture have not been universally selected, and the desired phenotypes can be achieved through several mutations on multiple loci [14–16], as is also the case with the *btr1* and *btr2* genes. There is, however, one sweep shared by all six groups, stretching over 245 kb of chromosome 1H (Fig. 5), which overlaps with the strongest selection signal detected through scanning a balanced supersample (Methods & Additional file 7: Fig. S5). This sweep has a distinct peak in the genomic window chr1H:544,978,515–545,595,921, which contains 12 high-confidence genes, including *SPA2* (see below). This short region is testimony of an initial phase of domestication shared by all extant cultivars, during which strong artificial selection brought about the first important domestication change.

Identification of the real selection targets from the selective sweeps of a species with low rates of effective recombination, such as barley, is challenging due to the size of the sweeps. Indeed, the regions we identified contain thousands of high-confidence genes (3437; 4356; 5406; 3671; 4554 and 5188 genes for the groups I–VI, respectively) hitch-hiking with perhaps a few dozen real targets. As a consequence, gene ontology analysis did not detect any over-represented biological or molecular functions. However, a search for signals of positive selection can be complemented with a search for the actual selected variants. If those selected variants are captured within the coding regions of the exome data, then by definition, they are expected to be non-silent (i.e. cause a change in the protein product), and to display contrasting frequencies in wild and domesticated barley. Variants were therefore categorized in the diversity matrix according to their position within genes, and for all sweeps in each group those non-synonymous variants and indels with the highest frequency departure from the wild superpopulation were identified. These variants (Table 2 & Additional file 9: Table S3) are the top candidates for the actual selection targets of barley domestication.

**Table 2:** Protein-changing variants with contrasting frequencies in wild and cultivated barley

Location (Morex V1)	Gene (Morex V1)	Gene (Morex V2)	Variant type	Variant in wild barley (frequency)	Variant in domesticated barley (frequency)	Variant in the 6ky barley	Groups where the position falls under a hard sweep	Significant BLASTP hit (species; query coverage; percent identity)
chr1H: 106920539	HORVU1 Hr1G024040	–	NS SNP	Ala (0.902)	Val (0.987)	Domesticated	I, II, III, V, VI, supersample	–
chr1H: 165877256	HORVU1 Hr1G029720	–	Indel	Wild-type transcripts .18 and .19 have premature stop codon (0.902)	transcripts .18 and 19 are 10 codons and 35 codons longer (0.981)	Missing data	I, III, V, VI, supersample	–
chr1H: 527209977	HORVU1 Hr1G081150	–	NS SNP	Ala (0.938)	Thr (0.986)	Wild-type	I, II, IV, supersample	–
chr1H: 545254976	HORVU1 Hr1G090080	HORVU.MOREX.r2.1 HG0074340	NS SNP	Arg (0.955)	Trp (0.995)	Domesticated	II, III, IV, V, VI, supersample	SPA2 (supressor of phyA-105) (*Arabidopsis*; 81%; 47.21%)
chr3H: 135052223	HORVU3 Hr1G029370	HORVU.MOREX.r2.3 HG0204760	NS SNP	Glu (0.951)	Asp (0.987)	Domesticated	IV	MRH1/MDIS2 (*Arabidopsis*; 97%; 44.93%)
chr5H: 592511492	HORVU5 Hr1G093710	–	NS SNP	Pro (0.906)	Leu (0.992)	Domesticated	I, V, VI	–
chr5H: 593178296	HORVU5 Hr1G093850	HORVU.MOREX.r2.5 HG0422940	NS SNP	Leu (0.911)	Val (0.982)	Domesticated	I, V, VI	Phototrophic-responsive NPH3 family protein (*Arabidopsis*; 97%; 44.99%)
chr7H: 540527349	HORVU7 Hr1G089090	–	NS SNP	Val (0.928)	Ala (0.995)	Domesticated	II, III	–
chr7H: 552413998	HORVU7 Hr1G090560	HORVU.MOREX.r2.7 HG0597530	Indel	wild-type C-terminus (0.996)	3-codon difference at the C-terminus (0.979)	Domesticated	II, III, V, VI	Cinnamyl alcohol dehydrogenase (*Triticum aestivum*; 98%; 92.37%)
chr7H: 552532125	HORVU7 Hr1G090580	HORVU.MOREX.r2.7 HG0597570	NS SNP	Leu (0.902)	Ile (0.990)	Domesticated	II, III, V, VI	–

Morex V1 refers to the barley genome assembly of Mascher et al. [19]; Morex V2 is the subsequent assembly of Monat et al. [32]

*Abbreviations*: *NS SNP* non-synonymous SNP. For domesticated barley variants, the frequency is > 0.9 in each of the six groups. Only BLASTP hits with > 20% query coverage and > 40% sequence identity against characterized proteins are reported

In the entire exome, there are only 10 protein-changing variants rare in the wild superpopulation but reaching fixation in all cultivated groups (Table 2 & Additional file 9: Table S3). Only four of these are found in genes whose products have good matches to well-described proteins. One is cinnamyl alcohol dehydrogenase (CAM), a crucial enzyme in phenylpropanoid biosynthesis, which in *Triticeae* plays a role in penetration resistance to pathogens such as *Blumeria graminis* [35] and *Rhizoctonia cerealis* [36]. Another gene, *MRH1/MDIS2*, is involved in root hair elongation [37] and potassium channel regulation [38]. Interestingly, two genes in this collection are involved in development of dark-grown seedlings. *SPA2*, whose strong selection signatures have been identified previously [9], encodes a potent repressor of photomorphogenesis in dark-grown seedlings in *Arabidopsis* [39], while the product of *NPH3* regulates phototrophic responses of etiolated seedlings to blue light [40]. This suggests that changes in seedling development, perhaps imposed by sowing seeds on tilled soil, were crucial for the early transformation of wild barley into an efficiently cultivated crop.

## Conclusion

By studying clearly defined genetic groups of domesticated barley, we provide clarity to our understanding of the population history of this crop. Inference of population splits and mixtures together with the analysis of selection sweeps jointly indicate a period of ancestry shared by all extant cultivated barley. We reveal that the founding population that emerged in the western Fertile Crescent underwent relatively little genetic selection, but that those changes that did occur affected traits involved in seedling emergence and pathogen resistance, indicating that these phenotypes should be considered alongside the classical ‘domestication traits’ such as loss of natural seed dispersal mechanisms and increases in seed size [41, 42]. During its expansion out of the western Fertile Crescent, the crop underwent regionally-specific episodes of gene flow and selection, giving rise to a modern genetic signature that has previously been interpreted as evidence of multiple domestications, but which we show can be rationalized with an origin from a single founding population as suggested by the genetics of the *Btr* loci. The strong, regional patterns of selection that operated outside of the Fertile Crescent have affected a wealth of loci whose future study could prove beneficial to improvement of the modern crop. Our results also highlight that group-specific selective sweeps might be generally important in crop evolution, rather than being relevant only when a crop displays distinct phenotypes or ecotypes [43, 44].

## Methods

### Data overview

The work combines data from three different sources. (1) Raw sequencing reads from 276 published exome capture libraries [10, 45] (174 landraces and improved cultivars, 102 wild barley accessions) were downloaded from the NCBI Sequence Read Archive (NCBI BioProjects PRJEB8044 and PRJEB1810) using fastq-dump command from the sratoolkit. (2) Similarly, published raw whole-genome data [18] from 10 seeds of 6000 years old domesticated barley found in the Yoram Cave (Israel) were downloaded (NCBI BioProject PRJEB12197). (3) Additionally, 46 exome capture libraries (27 wild barley accessions and 19 landraces) were prepared in our laboratory (NCBI BioProject PRJNA389721). The wild accessions were divided into four geographical populations based on their collection points: western Fertile Crescent (Israel, Jordan, Lebanon, and Syria and Turkey west of longitude 39.00), eastern Fertile Crescent (Iraq, Iran west of longitude 53.00, and Syria and Turkey east of longitude 39.00), Mediterranean (Cyprus, Greece and Libya), and Central Asia (Iran east of longitude 59.00, Afghanistan, Tajikistan, Turkmenistan and Uzbekistan). Additional details about the biological material are summarized in Additional file 1: Table S1.

### Preparation and sequencing of 46 barley exome capture libraries

DNA was extracted from a single dry seed per accession using a customized CTAB extraction protocol, followed by silica column-based purification. For the library preparation and exome capture, Technical Data Sheet for KAPA Library Preparation Kit (v1.14 and v2.11) and User’s Guide for NimbleGen SeqCap EZ Library SR (v4.2) were followed, with minor adjustments. For each accession, 1–5 μg DNA was sonicated in a Covaris S2 instrument, using intensity 4, 10% duty factor, 200 cycles/burst and 80–100 s treatment time. DNA concentration and fragment sizes were checked with nanodrop and 1% agarose gel electrophoresis. Fragment end repair, A-tailing and adapter ligation were performed with KAPA Library Preparation Kit and SeqCap Adapter Kit A (Roche), with MinElute kit (Qiagen) used for reaction clean-ups. Double size selection was performed with SPRIselect beads (Beckman Coulter Life Sciences), using 0.8–0.61 left-right ratio (sample: SPRIselect). Efficiency of size selection was checked by 1% agarose gel electrophoresis. Subsequently, the samples were amplified with KAPA HiFi HotStart ReadyMix and pre-LM-PCR Oligos 1 & 2, using 7 cycles and 58 °C annealing temperature. Following a reaction clean-up with High Pure PCR Purification Kit (Roche) the samples were measured with nanodrop and rechecked on a gel. In a pre-capture multiplex, 2–4 samples were mixed together in equal quantities to reach a combined mass of 1.2 μg. Multiplex Hybridization Enhancing Oligo Pool (SeqCap HE-Oligo Kit A; Roche) was added to the combined sample together with 5–10 μl of CapEZ Developer reagent, and the mixture was dried in a vacuum concentrator (Eppendorf) at 60 °C. The sample was hybridized with the barley exome capture design (Roche) at 47 °C for 72 h, using reagents from the SeqCap EZ Hybridization and Wash kit (Roche). Subsequent washing and recovery of the captured multiplex DNA samples were performed with the SeqCap EZ Pure Capture Bead Kit (Roche) according to the user’s guide. The post-LM-PCR was performed with the SeqCap EZ Accessory Kit v2 (Roche), using 14 cycles and 58 °C annealing temperature. The reaction was cleaned up according to the user’s guide, checked on a 1% agarose gel and measured with nanodrop. Each multiplexed sample was then sequenced on a single lane of Illumina HiSeq2500 (2 × 100 bp).

### Preparation of the genome-wide vcf file

All Illumina datasets were de-duplicated with tally [46], retaining the quality information. Adapter contamination and low-quality regions were subsequently removed with Trimmomatic [47], using the pair-end mode and the ILLUMINACLIP function, keeping only pairs where both trimmed reads are at least 25 nt long. The paired reads of the ancient DNA (aDNA) data sets (not the exomes) were subsequently merged into single reads with PEAR [48], discarding reads < 35 nt, and again de-duplicated with tally. Based on the amount of data and the coverage of the chloroplast genome, nine of the 10 aDNA datasets were considered substandard for the genome-wide genotyping and were excluded from the vcf production. The pre-processed exome dataset and one aDNA dataset (JK3014) were individually mapped onto the barley genome pseudomolecule assembly [19], using BWA-MEM in the smart pairing mode [49]. For this purpose, each of the seven barley reference chromosomes was split into two in order to circumvent the 512 Mb contig size constraint that would otherwise halt downstream bam indexing and disable the GATK pipeline. Following the read mapping, samtools and picard-tools were used for sorting, indexing and adding read groups in the individual bam files. Subsequently, HaplotypeCaller from the GATK4 package [50] was used to prepare individual gvcf files. The process was restricted by the -L flag to the coordinates of 80,553 genes (accounting for the changes caused by the splitting of the reference chromosomes) that comprise all mapped high- and low-confidence genes previously identified [19]. Individual gvcf files were combined into a single gvcf file using the CombineGVCFs walker from the GATK4 package, and subsequently, the combined gvcf file was genotyped with the GenotypeGVCFs walker. In the vcf output, the positions of the variants affected by the reference splitting were corrected and we refer to the resulting vcf file as the ‘complete’ dataset. This complete vcf file was further filtered using PLINK v1.90 [51] to produce the ‘base’ dataset (SNPs with ExcessHet< 3 and < 10% missing data points), and the ‘core’ dataset (linkage disequilibrium- [LD-] pruned biallelic SNPs from the base dataset). The stringent ExcessHet filter (sites with excess heterozygosity) was based on the expectation that barley, as a typically self-pollinating crop, has per site heterozygosities well-below the levels predicted by the Hardy-Weinberg (H-W) equilibrium. While the ExcessHet values (phred-scaled *p*-values of the exact test of the H-W equilibrium) of 75.9% of the sites are below 1, a distinct peak was observed just after the value 3 which predominantly corresponds to sites with singletons in a heterozygotic state. The LD pruning of the core dataset was performed in two steps: first, at *r*^*2*^ threshold 0.8, window size 10 kb, sliding step 1 SNP; and second, at *r*^*2*^ threshold 0.8, window size 50 SNPs, sliding step 1 SNP. Further manipulation and basic analysis of the vcf files (SNP density, depth of coverage, missingness, allelic frequency) were performed with PLINK and vcftools [52].

The exomes obtained by us have lower mean depth at the scored SNP sites compared to the exomes obtained from previous studies [10, 45] (13.5× and 23.4×, respectively), resulting in slightly higher proportion of missingness in our portion of the data (2.6% compared to 1.8% in the base dataset). However, this difference appears to have no effect on the genetic characterization of the subsets, as evidenced by the accession HOR4856 sequenced by us and Russell et al. [10] independently. Despite differing depth and missingness (Additional file 1: Table S1), these duplicated exomes are the closest neighbours in terms of identity-by-state (0.993 in the base and core datasets). There is also no correlation between missingness and the top eight PCs (all *p*-values > 0.15) that were used to define cultivated populations (see below).

### Population structure, splits and mixtures

PCA was performed on the core dataset (all samples) using smartpca from the Eigensoft package [53], without outlier removal. Based on perceived geographic barriers, the wild superpopulation was divided into four subpopulations: Mediterranean (Libya, Cyprus, Crete, Rhodes), western arm of the Fertile Crescent (west of the Euphrates), eastern arm of the Fertile Crescent (east of the Euphrates) and Central Asia (mainly Turkmenistan and Tajikistan). Based on the top two eigenvalues, several wild accessions, mostly originating outside the natural distribution range, were reclassified as hybrid/feral (noted in Additional file 1: Table S1). A separate PCA was conducted exclusively on the cultivated accessions, where six population genetic clusters (groups I–VI) were delineated along the top eight PCs (subsequent PCs often show significant correlation with missingness). Accessions were assigned to the groups I–VI based on PC thresholds (unsupervised approach in respect to geography), trying to maximize the number of assigned accessions along the minimum number of PCs (Fig. 1b–d). The PC3 and PC4 defined groups IV and V, respectively. The PC5 splits the group V into additional subgroups, however, this split was not considered in most subsequent analyses due to small sample sizes. The PC6 merely separates the accession FT380 previously classified as wild. The PC8 defined group VI. After the groups IV, V and VI were visualized along the top two axes of variation, PC1 was used to define group I. Finally, PC2, 3 and 8 jointly separated the groups II and III. Only 5% of accessions remained unassigned. The population assignment was checked for consistency on a Neighbor-Net network [20] constructed in SplitsTree4 using 1–IBS distance matrix calculated in PLINK, and also with sNMF ancestry coefficients that do not assume Hardy-Weinberg or linkage equilibria [21].

Joint allele frequency spectra were constructed using spreadsheet functions in Libre Office Calc. Splits and mixtures among the four wild populations and the cultivated groups I–VI were inferred with TreeMix 1.13 [22], using jackknife blocks of 1000 SNPs. The ABBA-BABA test was performed in Dsuite [25] using 50 jackknife blocks, the cultivated groups I–VI and the major, east-west split between the wild populations apparent from the Neighbor-Net graph (Additional file 2: Fig. S1). In the TreeMix and ABBA-BABA tests, *Hordeum bulbosum* was used as an outgroup. *H. bulbosum* assembly (NCBI BioProject PRJEB3403) was downloaded and shredded with GenomeTools into 300 bp fragments with 150 bp overlaps. Subsequently, the fragments were mapped onto the Morex pseudomolecule assembly, using bwa mem with -w 0 parameter. A bam file containing fragments with mapping quality > 10 (filtered with samtools) was imported to Geneious 6.1 (https://www.geneious.com) to build a fasta-formatted *H. bulbosum* consensus sequence corresponding to the Morex pseudomolecules. This fasta file was used with bedtools getfasta to obtain *H. bulbosum* data for each position within the base dataset. Subsequently, all biallelic positions from the base dataset with non-missing outgroup data (i.e. 1,437,134 SNPs) were used in TreeMix and Dsuite.

### Selective sweep detection

Highly uneven distribution of genes (and therefore also SNPs) across chromosomes in this exome dataset (Additional file 10; Fig. S6) has important implications for the detection of selective sweeps (described in detail in Additional file 6: Supplementary Note). After testing several strategies, a combination of two statistics was employed: first, the Diversity Reduction Index (DRI) calculated as π_WS_/π_DG_, where π_WS_ is the diversity in the wild superpopulation and π_DG_ is the diversity in the domesticated groups; and, second, Tajima’s D statistic, where the shift in the site-frequency spectrum is evaluated by comparing the total number of SNPs with the average number of nucleotide differences between pairs of sequences [54]. In the domestication context, DRI is often the clearest signal of selection, and several-fold reduction is typically required to consider a region as being swept [9, 43]. Tajima’s D measures a different signature of positive selection – excess of low-frequency variants – and the neutral model of evolution is usually rejected at values below − 2 [55]. The product of these two statistics was used for sweep detection, with a hard sweep threshold of −11.5, based on the joint distribution of the two statistics and the threshold performance on groups with different sample sizes (Additional file 6: Supplementary Note).

Subsequently, sweeps were detected in groups I–VI, as well as in the entire domesticated supersample. The domesticated supersample was created by randomly selecting 15 accessions from each group (i.e. 90 accessions in total) in order to avoid biases related to different group sizes. Ten random sampling iterations were performed. In all analyses, nucleotide diversity and Tajima’s D were calculated in sliding windows of 2000 SNPs and steps of 100 SNPs across the base dataset using VariScan-2.0.3 [56].

Inter-group overlaps of the observed sweeps were calculated with BEDtools [57] and the UpSet plot concept [34] was adapted for visualization. In order to test independence of the sweeps among groups, the stochastic distribution was modelled for each group by random placement of ‘sweeps’ of respective number and size across the chromosomes, followed by calculation of the resulting overlaps (10 million iterations). Significant excess of observed inter-group overlaps (i.e. non-independence of the sweeps) was then defined by quantiles of the modelled distribution.

### Functional annotation of the SNP data

An original Perl script was used to annotate the base dataset with additional information about the positions of the SNPs and their impact on the gene product. Using publicly available gtf annotations (https://webblast.ipk-gatersleben.de/barley_ibsc/downloads/), the position of each SNP was distinguished as either 5′–untranslated region, 3′–untranslated region, intron or coding sequence (CDS). SNP positions were recorded in all members of multiple overlapping transcript groups. For the CDS-located SNPs, synonymous and non-synonymous changes were distinguished, adding information about reference and alternative amino acids or stop codons. A collection of the non-synonymous SNPs was extracted and supplemented with all CDS-located indels from the complete data set. We refer to these non-synonymous SNPs and indels as ‘protein-changing’ variants. Candidate common domestication targets were identified as protein-changing variants with > 0.9 frequency in each cultivated group and < 0.1 frequency in the wild superpopulation. Each such position falls under a hard sweep in at least one of the groups, which indicates that such allelic distribution does not occur without selection. Since the common domestication targets were compiled irrespectively of the sweep information, this approach circumvents the problem of potential false negatives in the sweep detection procedure. Additionally, candidate selection targets were identified for each sweep in each group as the protein-changing variants with the highest frequency departure from the wild superpopulation.

## Supplementary Information

**Additional file 1: Table S1.** Barley accessions used in this study.

**Additional file 2: Figure S1.** Neighbor-Net network and its relation to the PCA-derived groups.

**Additional file 3: Figure S2.** sNMF ancestry coefficients and their relation to the PCA-derived groups.

**Additional file 4: Figure S3.** Haplotypes reconstructed from SNP data in a 400 kb region surrounding the *Btr1* and *Btr2* genes.

**Additional file 5: Figure S4.** Nucleotide diversity harboured in the wild populations.

**Additional file 6: Supplementary Note.** Detection of selective sweeps.

**Additional file 7: Figure S5.** Lack of universal selection targets during barley domestication.

**Additional file 8: Table S2.** Sweeping of previously identified domestication and improvement genes.

**Additional file 9: Table S3.** Protein-changing variants identified as possible selection targets for each sweep and each group.

**Additional file 10: Figure S6.** Distribution of SNPs and genes along the barley chromosomes.

## Data Availability

The raw sequencing reads and novel exome data supporting the conclusions of this article are available at NCBI Sequence Read Archive (https://trace.ncbi.nlm.nih.gov/Traces/sra/sra.cgi?; accession numbers listed in Additional file 1: Table S1) and at NCBI BioProject PRJNA389721 (https://www.ncbi.nlm.nih.gov/bioproject/?term=PRJNA389721), respectively. The base and core datasets, as well as the vcf file with functionally annotated SNPs, are available at Mendeley Data (10.17632/BBV2MDXWCR.1). The Perl script used to annotate the base dataset is available at gitlab (https://gitlab.com/davidarmisen/classification-of-snps-within-primary-transcript/). The following previously published datasets were used: NCBI BioProject PRJEB8044 (https://www.ncbi.nlm.nih.gov/Traces/study/?acc=ERP009079&o=acc_s%3Aa), PRJEB1810 (https://www.ncbi.nlm.nih.gov/Traces/study/?acc=ERP002487&o=acc_s%3Aa), PRJEB12197 (https://www.ncbi.nlm.nih.gov/Traces/study/?acc=ERP013644&o=acc_s%3Aa), PRJEB3403 (https://www.ncbi.nlm.nih.gov/assembly/GCA_900070015.1#/def).

## Abbreviations

6ky: 6000 years old
aDNA: Ancient DNA
CDS: Coding sequence
DNA: Deoxyribonucleic acid
DRI: Diversity reduction index
H-W: Hardy-Weinberg
IBS: Identity-by-state
kb: Kilobase pair
LD: Linkage disequilibrium
MB: Megabase pair
NCBI: National Center for Biotechnology Information
PC: Principal component
PCA: Principal component analysis
PCR: Polymerase chain reaction
SNP: Single nucleotide polymorphism
