# Supplementary material for: Episodes of gene flow and selection during the evolutionary history of domesticated barley
Source: BMC Genomics. 2021 Apr 1;22:227. doi: 10.1186/s12864-021-07511-7 (PMC8015183; doi:10.1186/s12864-021-07511-7)
Supplement: Supplementary file 2 — Additional file 2: Figure S1. Neighbor-Net network and its relation to the PCA-derived groups. [file 12864_2021_7511_MOESM2_ESM.pdf]

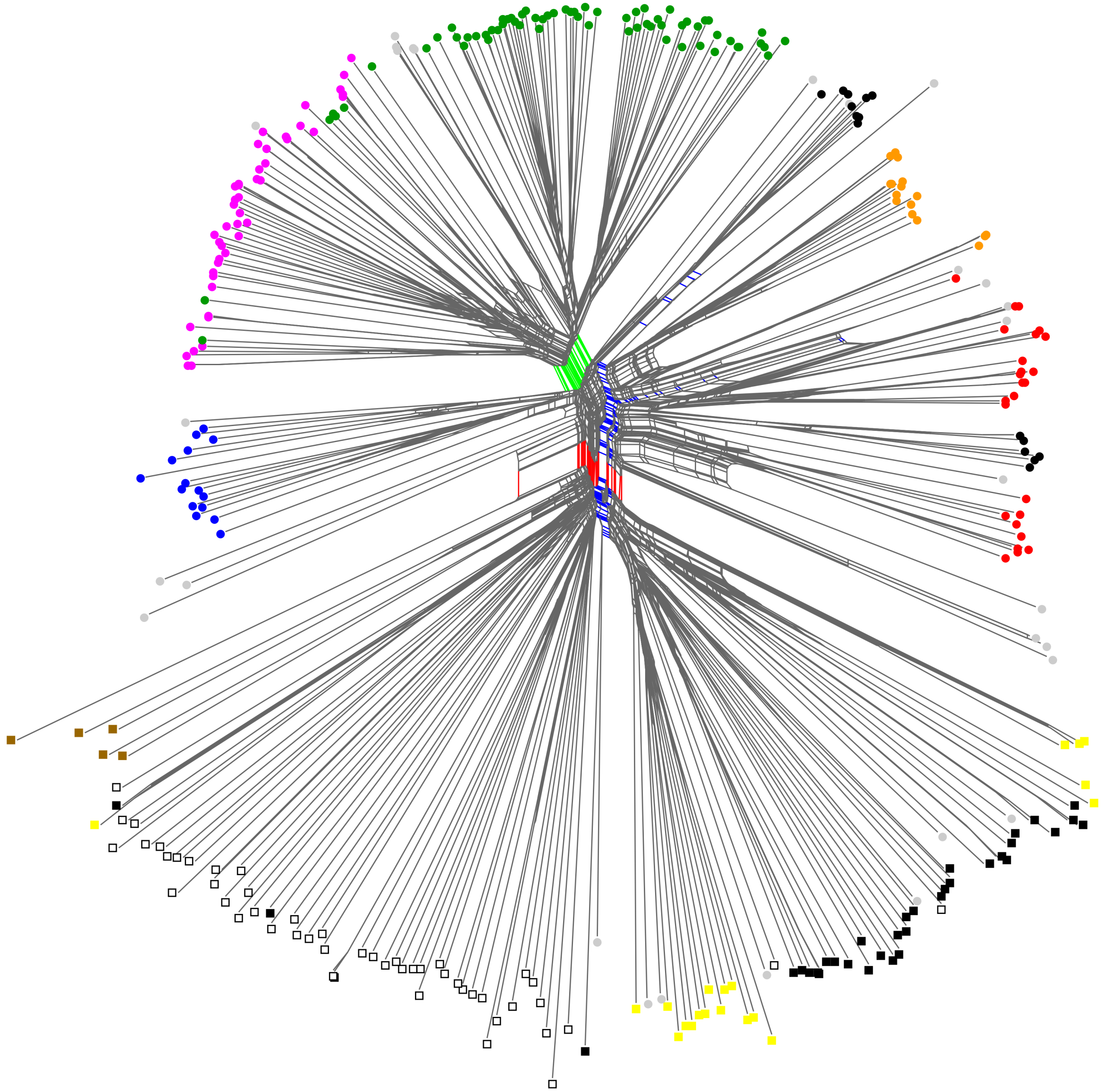

Fig. S1 Neighbor-Net network and its relation to the PCA-derived groups. Nodes are marked with circles for cultivated accessions (coloured as in Fig. 1) and squares for wild accessions (white, western Fertile Crescent; black, eastern Fertile Crescent; brown, Mediterranean; yellow, Central Asia). Edges separating wild and domesticated barley (i.e. edges grouping all domesticated barley together) are highlighted in red. Edges clustering groups I, V and VI with the eastern wild populations are highlighted in blue. Edges separating groups II and III are highlighted in green.
