# Supplementary material for: Episodes of gene flow and selection during the evolutionary history of domesticated barley
Source: BMC Genomics. 2021 Apr 1;22:227. doi: 10.1186/s12864-021-07511-7 (PMC8015183; doi:10.1186/s12864-021-07511-7)
Supplement: Supplementary file 3 — Additional file 3: Figure S2. sNMF ancestry coefficients and their relation to the PCA-derived groups. [file 12864_2021_7511_MOESM3_ESM.pdf]

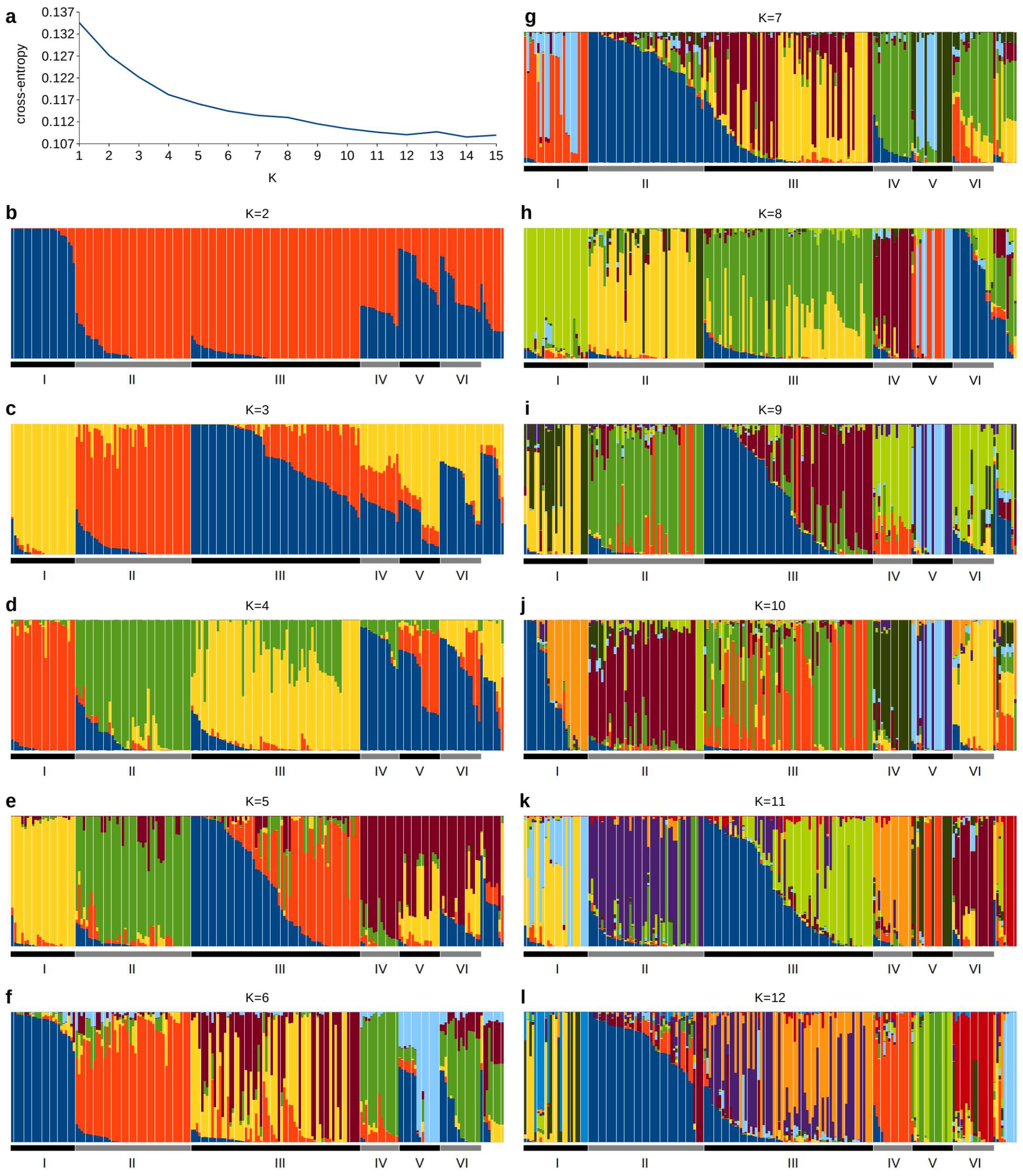

Fig. S2 sNMF ancestry coefficients and their relation to the PCA-derived groups. (a) Cross-entropy for 1–15 populations (K). (b–l) Ancestry coefficients for all cultivated accessions (horizontal bars) obtained for 2–12 populations, and their relation to the PCA-derived groups indicated on the X-axis. At K=4, groups I–III are well differentiated, but the remaining groups IV–VI form one population (blue) with various degrees of admixture. At the higher values of K, group V is distinguished and its two subgroups are apparent. However, ancestry of group III becomes more complex, and group VI is only distinguished at K>7.
