# Supplementary material for: Episodes of gene flow and selection during the evolutionary history of domesticated barley
Source: BMC Genomics. 2021 Apr 1;22:227. doi: 10.1186/s12864-021-07511-7 (PMC8015183; doi:10.1186/s12864-021-07511-7)
Supplement: Supplementary file 4 — Additional file 4: Figure S3. Haplotypes reconstructed from SNP data in a 400 kb region surrounding the Btr1 and Btr2 genes. [file 12864_2021_7511_MOESM4_ESM.pdf]

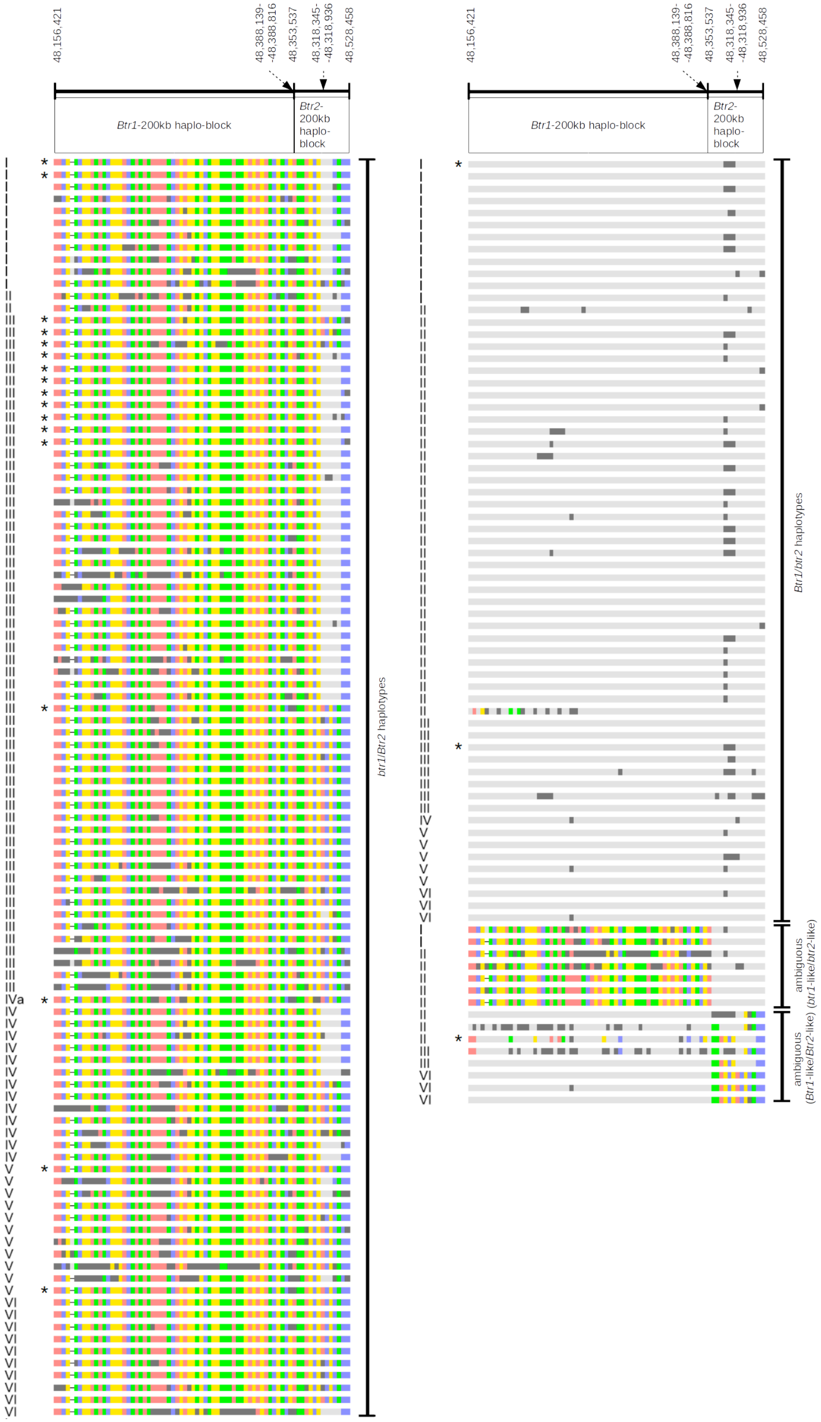

Fig. S3 Haplotypes reconstructed from SNP data in a 400 kb region surrounding the *Btr1* and *Btr2* genes. The *Btr1* and *Btr2* genes were not included in the barley exome design, so we do not have direct data from the causal non-brittle mutation sites. To address this problem the surrounding haplotypes were reconstructed, which in most cases allows inference about the *Btr1-Btr2* genotypes, as some of them are known from previous publications (marked with an asterisk). The haplotypes are aligned to the Morex V1 reference (*Btr1/btr2*) and disagreements to the reference nucleotides are highlighted in colour. Ambiguities and missing data are shaded dark grey. For each haplotype, the group of origin is indicated on the left. 'IVa' marks the 6ky barley haplotype. The distribution of the haplotypes across the groups suggests that the *btr1* and *btr2* mutations occurred in groups IV and II, respectively. The remaining groups contain a mixture of the two haplotypes that does not deviate from random distribution. The alignment was visualized in Geneious 11.1.5 (https://www.geneious.com).
