# Supplementary material for: Episodes of gene flow and selection during the evolutionary history of domesticated barley
Source: BMC Genomics. 2021 Apr 1;22:227. doi: 10.1186/s12864-021-07511-7 (PMC8015183; doi:10.1186/s12864-021-07511-7)
Supplement: Supplementary file 5 — Additional file 5: Figure S4. Nucleotide diversity harboured in the wild populations. [file 12864_2021_7511_MOESM5_ESM.pdf]

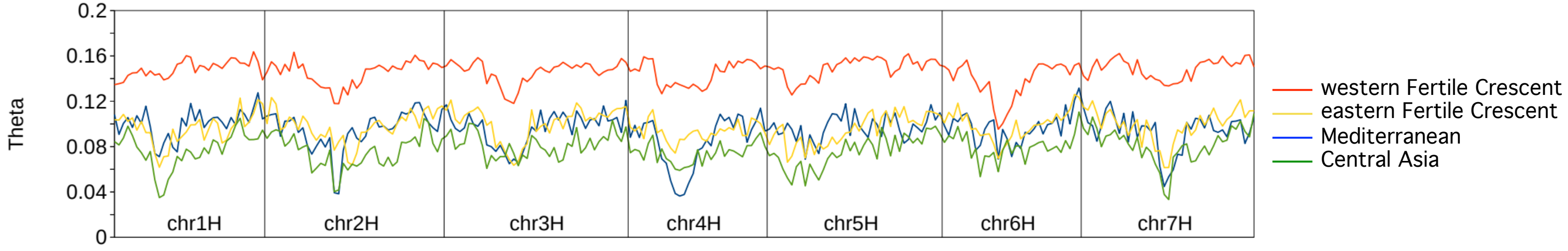

Fig. S4 Nucleotide diversity in the wild populations. Theta was calculated in windows of 10,000 SNPs across the base dataset.
