# Supplementary material for: Episodes of gene flow and selection during the evolutionary history of domesticated barley
Source: BMC Genomics. 2021 Apr 1;22:227. doi: 10.1186/s12864-021-07511-7 (PMC8015183; doi:10.1186/s12864-021-07511-7)
Supplement: Supplementary file 6 — Additional file 6: Supplementary Note. Detection of selective sweeps. [file 12864_2021_7511_MOESM6_ESM.docx]

**Supplementary Note** – Detection of selective sweeps

Positive selection is the central force in the process of domestication and adaptation to new environmental conditions. Strong positive selection applied on a genetic variant with low initial population frequency results in a selective sweep, and generally leaves three kinds of genomic signatures that can be used for identification of the loci affected by the selection [1,2]. First, as the frequency of the selected variant increases in the population, linked variation diminishes due to the genetic hitch-hiking effect [3]. This decrease of variation is easily detectable by comparing the nucleotide diversity (π) of the studied population to a reference population, which in the domestication context means comparing the diversity of the domesticated and wild populations. Second, positive selection causes a shift in the site frequency spectrum (SFS), creating a local excess of low- and high-frequency variants and a lack of medium-frequency variants. This shift in the SFS can be detected by relatively simple summary statistics such as Tajima’s D [4], where the average number of nucleotide differences between pairs of sequences is compared to the total number of segregating sites, but more sophisticated methods have been developed. A frequently used tool SweeD [5] uses a composite-likelihood-ratio (CLR) test to compare the probability of the observed polymorphism data under the standard neutral model with the probability of observing the data under a selective sweep model. Finally, selective sweeps can be also identified from a particular pattern of linkage disequilibrium (LD). A popular implementation of this approach, OmegaPlus [6], identifies selected loci as genomic points where pairs of variants on each side show elevated LD, but pairs of variants across the point show low LD.

A selective sweep is expected to contain all three signatures of positive selection. However, in practice, genomic scans of different signatures often yield inconsistent results [7,8]. The inconsistencies likely stem from the fact that the three signatures of selection are not robust to demographic factors ‒ e.g. the SFS shift towards the low-frequency variants can occur as a result of population expansion [9]. Detection of positively selected genes therefore remains challenging, prompting us to test the performance of several approaches with our data.

The SFS-based program SweeD [5], when used on the cultivated groups without analytical SFS calculation (i.e. when the background SFS is calculated from the data), did not identify any selective sweeps (CLR=0 for all grid points). Similarly, Russell et al. [7] did not find any strong selection signals when SweeD was used with a genome-wide SFS as the background model. This could be related to the fact that the majority of SNPs in the barley exome data are rare, and the excess of low-frequency variants is even more pronounced in wild barley (i.e. in the population with no history of artificial selection) [9]. Since our diversity data set is largely based on the sequence data produced by Russell et al. [7], we refrained from further testing of the SweeD program.

The LD-based program OmegaPlus [6] detected many strong selection signals and the ω statistic exhibited weak correlation with the diversity reduction index (DRI) (Table N1). However, we also noticed that ω often peaks at the start and end of longer sweeps (Fig. N1), and we consider this to be an artefact likely caused by a hard limit for a sweep length (-maxwin), which appears to be 4,999 SNPs in OmegaPlus and is insufficient for self-pollinating species with very low levels of effective recombination. We have also tested the RAiSD program [10], which quantifies all three signatures of selection in a composite evaluation test. Unfortunately, RAiSD is unsuitable for exome-based datasets due to a high correlation of the μ statistic with the distance from the centromere. The correlation coefficient is between –0.624 and –0.765 per chromosome, and is mainly determined by the μ_var component of μ (Fig. N2). The strong correlation is caused by the uneven distribution of SNPs across chromosomes in exome data (Additional file 10, Fig. S6), which is contrary to the assumption employed for μ computation.

**Table N1** Correlation of different sweep-detection statistics across the whole genome

| Genetic group | ω and log(DRI) | ω and Tajima’s D | log_2_(DRI) and Tajima’s D |
| --- | --- | --- | --- |
| I | *r* = 0.4238 | *r* = –0.2229 | *r* = –0.6946 |
| II | *r* = 0.3337 | *r* = –0.1345 | *r* = –0.6656 |
| III | *r* = 0.1946 | *r* = –0.0745 | *r* = –0.7423 |
| IV | *r* = 0.5427 | *r* = –0.2064 | *r* = –0.5384 |
| V | *r* = 0.3527 | *r* = –0.1925 | *r* = –0.5533 |
| VI | *r* = 0.4596 | *r* = –0.2053 | *r* = –0.6021 |

**
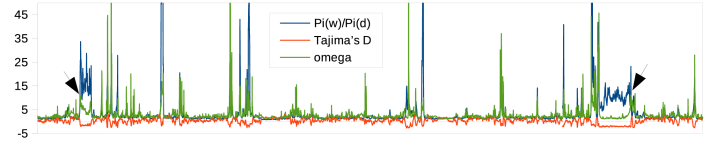
**

**Fig. N1** Scan of several sweep-detection statistics in group I. The horizontal axis shows concatenated chromosomes 1H–4H; the vertical axis shows the values of both statistics. The arrowheads point at two cases where the DRI and Tajima’s D indicate a long selective sweep, but ω peaks at the borders of those sweeps. OmegaPlus was run with -minwin 800, -maxwin 40000 and grid points adjusted to match the sliding windows of Tajima’s D and DRI.


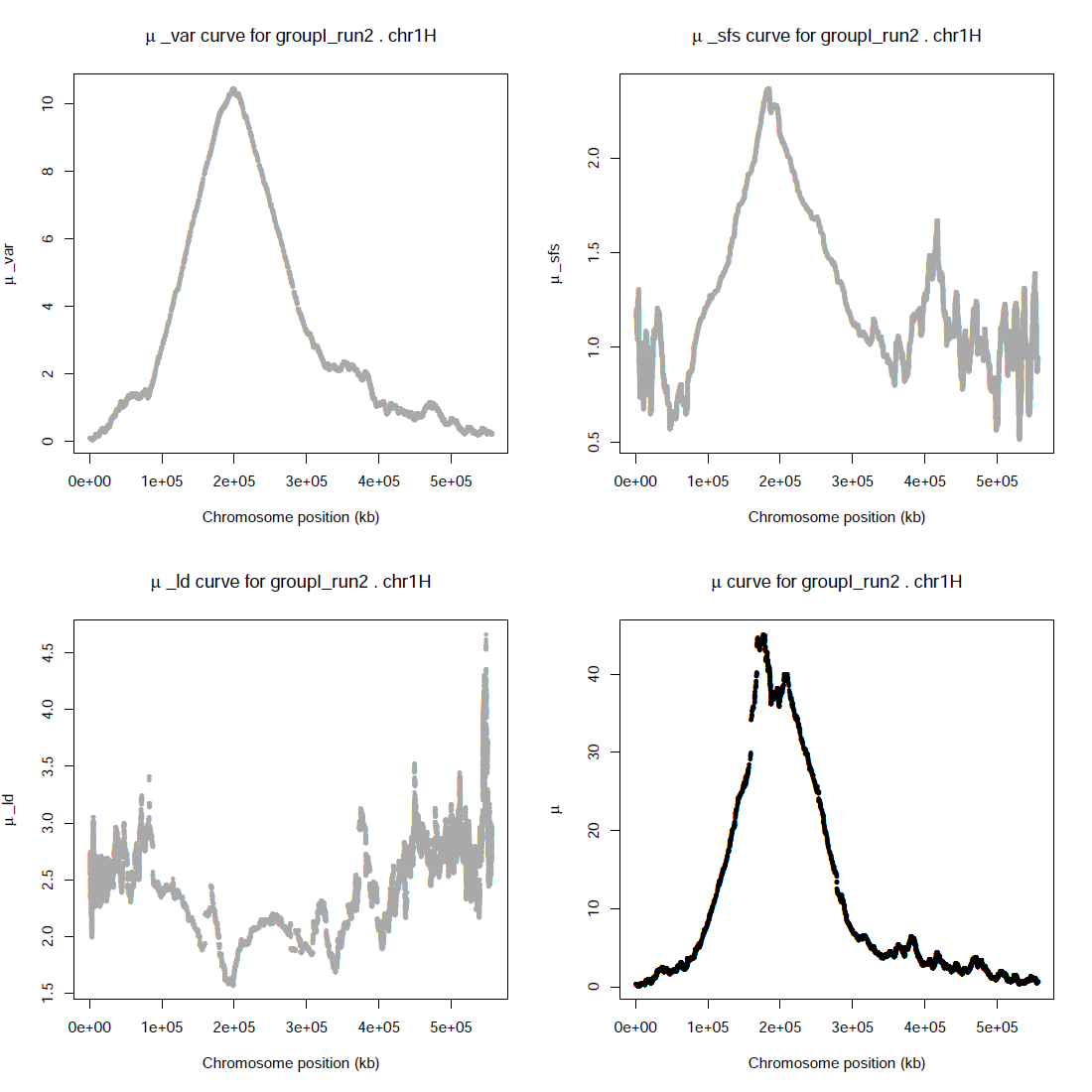


**Fig. N2** RAiSD results for chromosome 1H in group I. The values of μ_var and µ_sfs are negatively correlated with the distance from the centromere, while μ_ld shows weaker positive correlation. The composite value μ is therefore unsuitable for sweep detection in exome data. RAiSD was run with the following parameters: -M 1 -w 2000 -c 2 -P -A 0.995.

Due to these issues, we decided to base the sweep detection on relatively simple, but widely used summary statistics ‒ Tajima’s D and DRI ‒ which have relatively high negative correlation in our data (Table N1), indicating consistency for evaluating selection. Both were calculated from a fixed number of SNPs (2000 SNP windows; 100 SNPs sliding step) rather than from windows with fixed length. This approach is preferable with exome data, where a 1 Mb window in the centromeric region may contain no SNPs, while a window of the same size in the telomeric region often contains thousands of SNPs. Joint frequency distribution of Tajima’s D and DRI (Fig. N3) shows that the inverse relationship is particularly clear at low values of Tajima’s D and high diversity reduction (i.e. the two statistics correspond well at values associated with selection).


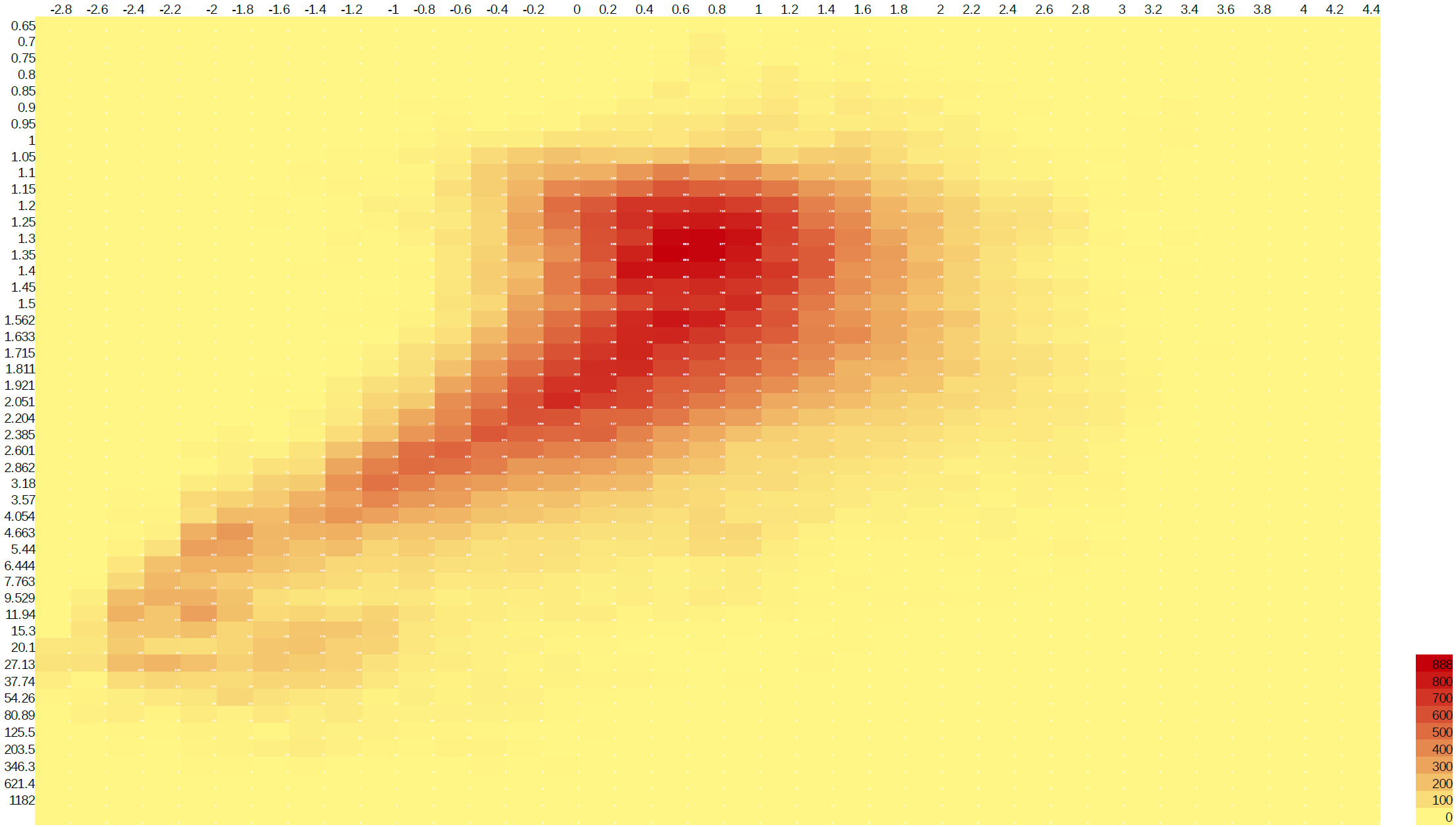


**Fig. N3** Joint frequency distribution of Tajima’s D and DRI values for all examined windows in groups I–VI. For each genomic window, Tajima’s D and DRI were calculated. The heatmap indicates the number of windows that fall under the specified categories (Tajima’s D categories along the horizontal axis, DRI categories along the vertical axis). For better visualization, the DRI categories change linearly up to a value of 1.5, and exponentially from 1.5 onwards. The bottom-left tail corresponds to selective sweeps.

Subsequently, we searched for an optimal threshold for classifying genomic windows as selective sweeps. First, we checked whether stochastic effects can create false signals of selection in randomly selected groups with small sample size. We created five subsamples of 15 accessions picked randomly from the wild superpopulation, and performed genome-wide scans of Tajima’s D and DRI (Fig. N4). In total, only 0.1% of the genomic windows showed 2-fold or higher reduction in nucleotide diversity, compared to the wild supersample, and only 0.2% of the windows showed Tajima’s D <–2, which is the standard threshold for rejecting neutral evolution. Only 0.004% of the genomic windows were classified as swept according to our threshold (DRI*Tajima’s D) <–11.5 (see below). Strictly speaking, this


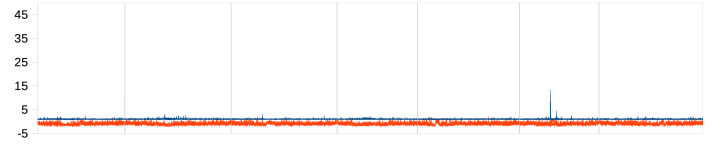


**Fig. N4** Genome-wide scans of Tajima’s D (red) and DRI (blue) performed on random samples of 15 wild accessions. The horizontal axis shows concatenated chromosomes 1H–7H separated by vertical lines; the vertical axis shows the values of both statistics. Results of five sampling iterations are overlaid.

indicates that in a random sample of 15 individuals, zero (or a very few) strong signals of selection should normally be detected, and therefore, our groups I–VI (with dozens of strong sweeps in each) are not random assemblages in respect to selection.

Finally, we searched for a sweep detection threshold that takes into account both statistics and has the optimal specificity (the ratio of correctly identified negatives to real negatives) and sensitivity (the ratio of correctly identified positives to real positives). We tested specificity and sensitivity for a range of thresholds (DRI*Tajima’s D) by comparing the detection results from various subsamples of the largest cultivated group III to the ‘real’ detection result of the full group III (66 accessions). The specificity and sensitivity was determined for subsamples of 15, 25 and 45 accessions (10 random sampling iterations for each sample size) (Fig. N5). Since both measures of performance are negatively influenced by low sample size, we picked the threshold according to maximum sensitivity at n=15 (–11.5). When this threshold is applied on samples of 15 accessions, 3.3% of the ‘no-sweep’ windows are incorrectly detected as sweeps, and ~16.1% of the real sweeps are missed. The performance is better on samples of 45 accessions, where <1% of the ‘no-sweep’ windows are incorrectly detected as sweeps and <5% of the real sweeps are missed. Theoretically, the dynamic threshold (DRI*Tajima’s D) <–11.5 is met only when Tajima’s D is negative (indicating excess of rare variants) and the reduction of diversity is several-fold. When applied to the real data in the six domesticated barley groups, the threshold yielded a collection of windows where the nucleotide diversity is at least 4.6× lower compared to the wild superpopulation, and Tajima’s D is always below –0.12.

**
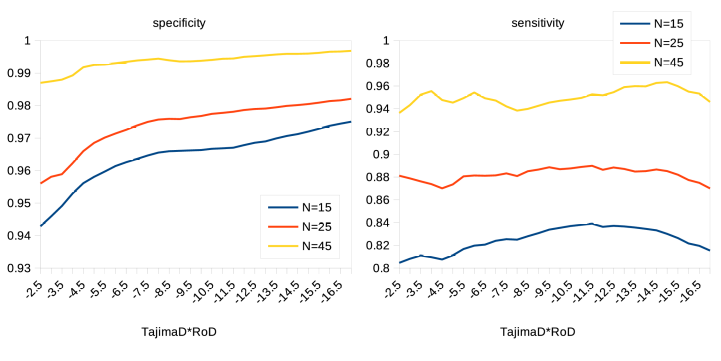
**

**Fig. N5** Specificity and sensitivity of sweep detection at various (DRI*Tajima’s D) thresholds.

**References**

1. Nielsen R. Molecular signatures of natural selection. Annu Rev Genet. 2005; 39: 197–218.

2. Pavlidis P, Alachiotis N. A survey of methods and tools to detect recent and strong positive selection. J Biol Res (Thessalon). 2017; 24: 7.

3. Smith JM, Haigh J. The hitch-hiking effect of a favourable gene. Genet Res. 1974; 23: 23–35.

4. Tajima F. Statistical method for testing the neutral mutation hypothesis by DNA polymorphism. Genetics. 1989; 123: 585–95.

5. Pavlidis P, Živković D, Stamatakis A, Alachiotis N. SweeD: likelihood-based detection of selective sweeps in thousands of genomes. Mol Biol Evol. 2013; 30: 2224–34.

6. Alachiotis N, Stamatakis A, Pavlidis P. OmegaPlus: a scalable tool for rapid detection of selective sweeps in whole-genome datasets. Bioinformatics. 2012; 28: 2274–5.

7. Russell J, Mascher M, Dawson IK, Kyriakidis S, Calixto C, Freund F, Bayer M, Milne I, Marshall-Griffiths T, et al. Exome sequencing of geographically diverse barley landraces and wild relatives gives insights into environmental adaptation. Nature Genet. 2016; 48: 1024–30.

8. Pankin A, Altmüller J, Becker C, von Korff M. Targeted resequencing reveals genomic signatures of barley domestication. New Phytol. 2018; 218: 1247–59.

9. Keinan A, Clark AG. Recent explosive human population growth has resulted in an excess of rare genetic variants. Science. 2012; 336: 740–3.

10. Alachiotis N, Pavlidis P. RAiSD detects positive selection based on multiple signatures of a selective sweep and SNP vectors. Commun Biol. 2018; 1: 79.
