# Supplementary material for: Episodes of gene flow and selection during the evolutionary history of domesticated barley
Source: BMC Genomics. 2021 Apr 1;22:227. doi: 10.1186/s12864-021-07511-7 (PMC8015183; doi:10.1186/s12864-021-07511-7)
Supplement: Supplementary file 7 — Additional file 7: Figure S5. Lack of universal selection targets during barley domestication. [file 12864_2021_7511_MOESM7_ESM.pdf]

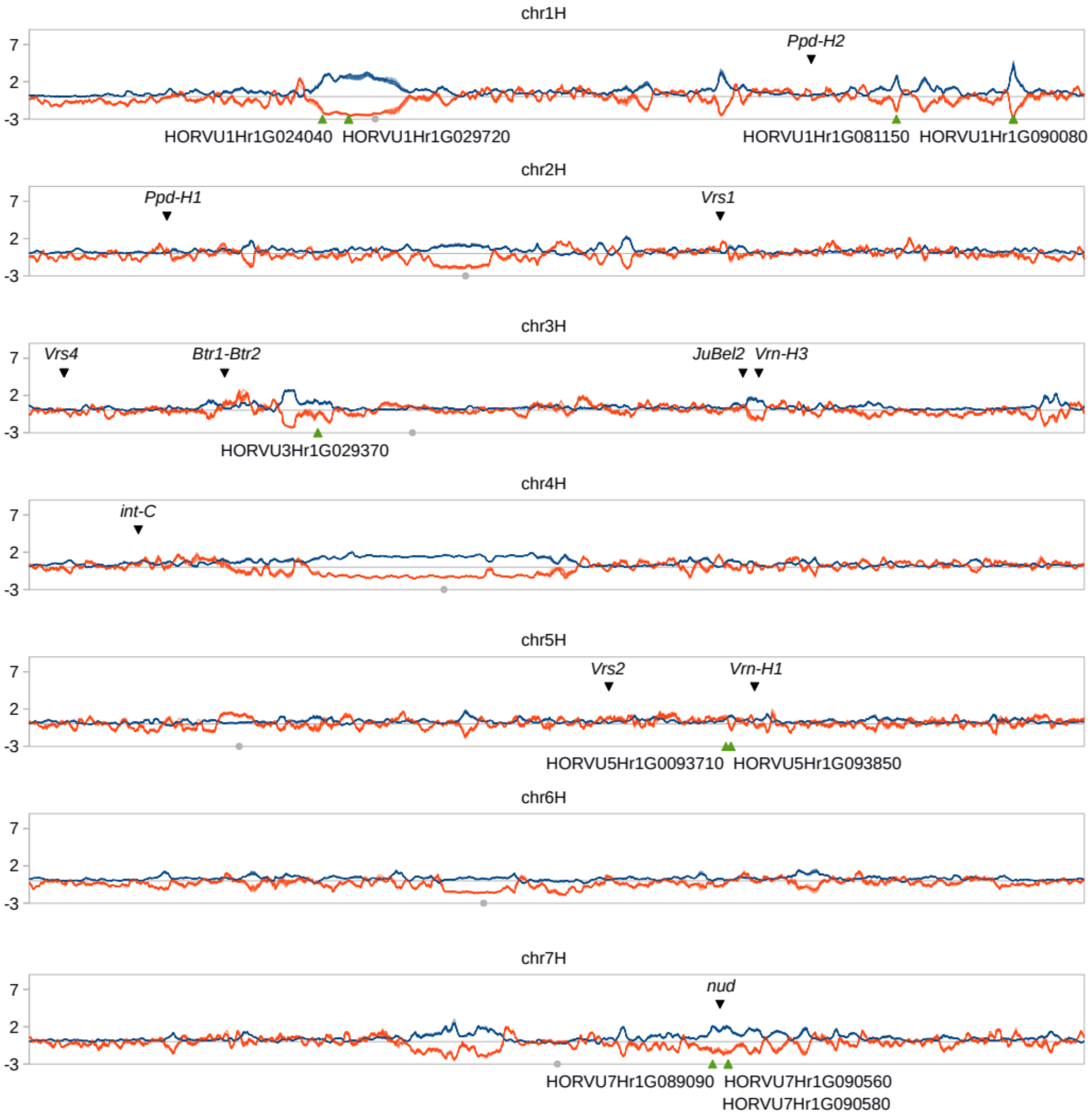

Fig. S5 Lack of universal selection targets during barley domestication. The blue and red curves represent the $\log_2$(DRI) and Tajima's D scans for a balanced supersample, respectively. Centromeres are indicated with full grey circle. When a supersample is created by balanced sampling of groups I–VI (15 accessions picked randomly from each group; the results of ten sampling iterations overlaid), most of the selection signals disappear. This confirms that the relative scarcity of sweeps overlapping between groups is not caused by false negatives in the group-specific profiles, and demonstrates the rarity of universal selection targets during barley domestication. Positions of previously described domestication genes are indicated with black arrowheads and do not correspond to clear selection signals. The green arrowheads indicate positions of protein-changing variants with low frequency (p<0.1) in the wild superpopulation and high frequency (p>0.9) in each cultivated group (and consequently, p>0.97 in the cultivated superpopulation), which in some cases correspond to clear selective sweeps. Further information on these variants is given in Table 2.
