# Supplementary material for: Episodes of gene flow and selection during the evolutionary history of domesticated barley
Source: BMC Genomics. 2021 Apr 1;22:227. doi: 10.1186/s12864-021-07511-7 (PMC8015183; doi:10.1186/s12864-021-07511-7)
Supplement: Supplementary file 10 — Additional file 10: Figure S6. Distribution of SNPs and genes along the barley chromosomes. [file 12864_2021_7511_MOESM10_ESM.pdf]

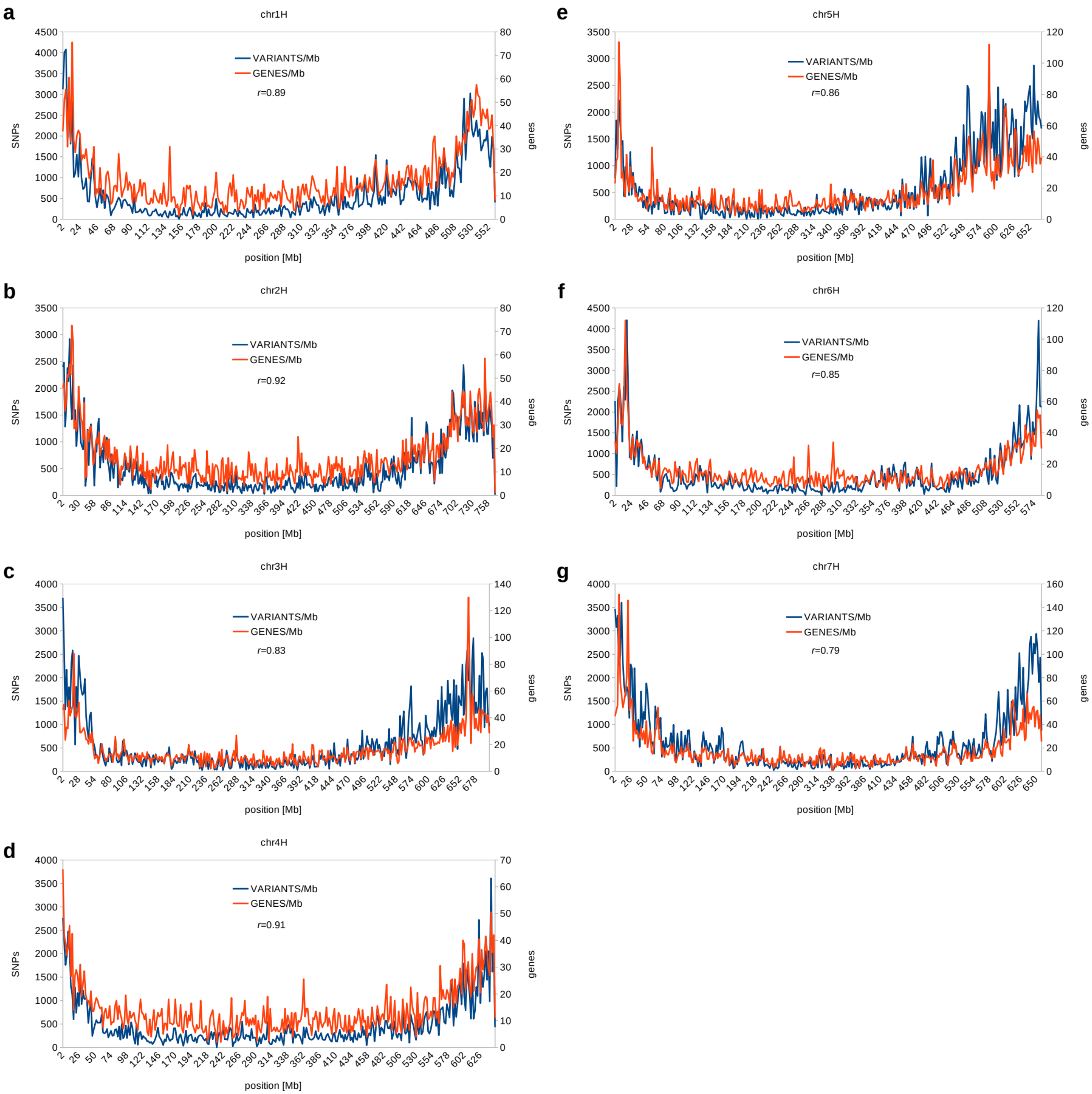

Fig. S6 Distribution of SNPs and genes along the barley chromosomes. SNP and gene densities were calculated in 2 Mb windows for chromosomes 1H–7H. SNPs from the 'base' dataset and all mapped high- and low-confidence genes were used for the calculations. The SNPs are concentrated at the chromosomal ends and scarce in the centromeric regions, but this is largely due to the distribution of genes, which follows the same pattern (correlation coefficients are shown in the legends). This means that the exome approach does not collect data evenly across chromosomes, and this unevenness must be accounted for in design of the sweep detection strategy (see Additional file 6: Supplementary Note).
